# The Role of Substrate Mediated Allostery in the Catalytic Competency of the Bacterial Oligosaccharyltransferase PglB

**DOI:** 10.3389/fmolb.2021.740904

**Published:** 2021-09-15

**Authors:** Brittany R. Morgan, Francesca Massi

**Affiliations:** Department of Biochemistry and Molecular Pharmacology, University of Massachusetts Medical School, Worcester, MA, United States

**Keywords:** molecular dynamics, substrate binding, consensus sequence specificity, N-glycosylation efficiency, allosteric communication

## Abstract

The oligosaccharyltransferase of *Campylobacter lari* (PglB) catalyzes the glycosylation of asparagine in the consensus sequence N-X-S/T, where X is any residue except proline. Molecular dynamics simulations of PglB bound to two different substrates were used to characterize the differences in the structure and dynamics of the substrate-enzyme complexes that can explain the higher catalytic efficiency observed for substrates containing threonine at the +2 position rather than serine. We observed that a threonine-containing substrate is more tightly bound than a serine-containing substrate. Because serine lacks a methyl group relative to threonine, the serine-containing peptide cannot stably form simultaneous van der Waals interactions with T316 and I572 as the threonine-containing substrate can. As a result, the peptide-PglB interaction is destabilized and the allosteric communication between the periplasmic domain and external loop EL5 is disrupted. These changes ultimately lead to the reorientation of the periplasmic domain relative to the transmembrane domain such that the two domains are further apart compared to PglB bound to the threonine-containing peptide. The crystal structure of PglB bound to the peptide and a lipid-linked oligosaccharide analog shows a pronounced closing of the periplasmic domain over the transmembrane domain in comparison to structures of PglB with peptide only, indicating that a closed conformation of the domains is needed for catalysis. The results of our studies suggest that lower enzymatic activity observed for serine versus threonine results from a combination of less stable binding and structural changes in PglB that influence the ability to form a catalytically competent state. This study illustrates a mechanism for substrate specificity via modulation of dynamic allosteric pathways.

## Introduction

Asparagine-linked glycosylation (N-glycosylation) is an essential function in eukaryotes (Helenius and Aebi, 2004; Mohorko et al., 2011; Aebi, 2013). The attachment of a hydrophilic glycan changes the chemical properties of the protein, conferring new interactions and potentially affecting folding (Mohorko et al., 2011). N-glycosylation is also involved in quality control: it provides information about folding status and thus can target misfolded proteins for degradation (Helenius and Aebi, 2004; Aebi, 2013). In prokaryotes, N-glycosylation is non-essential but plays important roles such as in the extremophile abilities of archaea (Eichler, 2013) and in the pathogenesis of bacteria (Szymanski et al., 2002; Nothaft and Szymanski, 2013).

N-glycosylation is catalyzed by oligosaccharyltransferase (OST), which attaches a lipid-linked oligosaccharide (LLO) to the consensus sequence N-X-S/T of the acceptor substrate (where X is any amino acid except proline) (Aebi, 2013). OST is usually an oligomeric membrane protein complex in eukaryotes (Kelleher and Gilmore, 2006). In mammals, OST consists of seven subunits: ribophorin I, DAD1, N33/IAP, OST4, STT3A/B, Ost48, and ribophorin II (Kelleher and Gilmore, 2006); in prokaryotes, OST is a single subunit homologous to the catalytic STT3 eukaryotic subunit (Szymanski and Wren, 2005; Eichler, 2013). The structural homology with the eukaryotic STT3 is strongest for *Campylobacter jejuni* OST and it is thought to have a similar catalytic mechanism (Wacker et al., 2002; Kelleher and Gilmore, 2006; Li et al., 2010). The bacterial OST from *C. jejuni* and its homolog from *C. lari* have been studied to understand the fundamentals of N-glycosylation because of the strong homology and their relative simplicity (Wacker et al., 2002; Li et al., 2010; Jaffee and Imperiali, 2011; Ihssen et al., 2012; Gerber et al., 2013; Lizak et al., 2014; Barre et al., 2017). These studies have identified conserved motifs and catalytically important residues. A critical step forward in understanding how these features work together was provided by the crystal structure of the full-length *C. lari* OST (commonly referred to as PglB) bound to an acceptor substrate peptide, which provided structural insights into the catalytic mechanism and the role of conserved motifs (Lizak et al., 2011). Recent structures of eukaryotic OST complexes, which elucidate the interactions between different subunits, further support the structural homology between the catalytic STT3 subunit and *C. lari* PglB (Bai et al., 2018; Braunger et al., 2018; Ramírez et al., 2019). Due to the size and complexity of the eukaryotic OSTs, the homologous bacterial OSTs continue to be useful model systems to understand common features of N-glycosylation.

**FIGURE 1:**
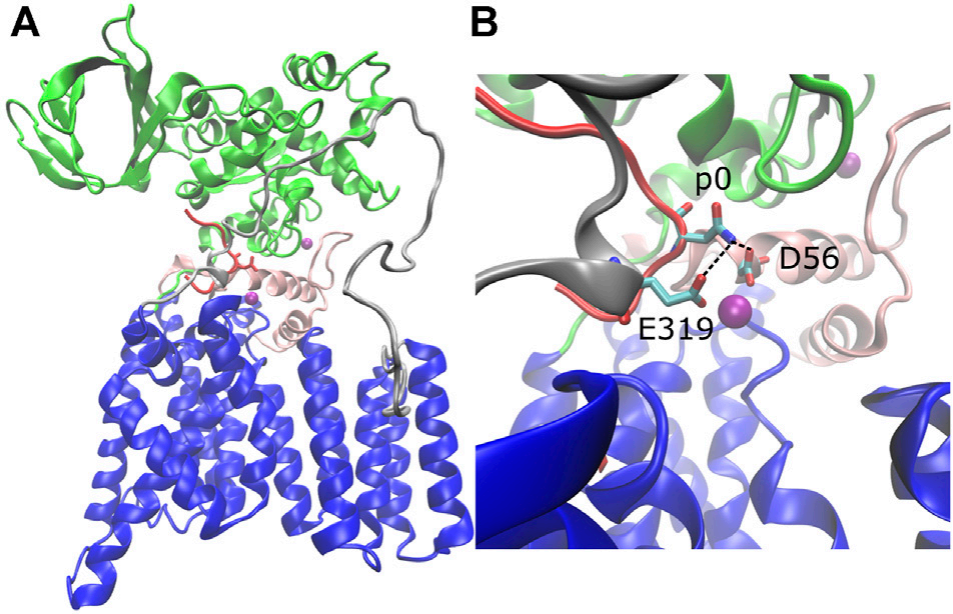
Structure of *C. lari* PglB and catalytically relevant hydrogen bonds. **(A)** Crystal structure of PglB bound to the peptide sequence GDQNATFG (PDB code: 3RCE) with missing loops modeled. Blue: transmembrane domain, green: periplasmic domain, red: acceptor substrate peptide with acceptor asparagine side chain shown, pink: external loop EL1, gray: external loop EL5, purple: Mg^2+^ ions. **(B)** Enlarged view of catalytic pocket showing catalytically relevant H-bond pairs of E319 and D56 with the acceptor asparagine (p0) colored by atom type: C (cyan), N (blue), and O (red).

The structure of *C. lari* PglB is composed of a transmembrane domain and a periplasmic domain (Lizak et al., 2011). The transmembrane domain (residues 1–432) consists of thirteen transmembrane helices (Figure 1A, blue) connected by short cytoplasmic or external loops and two long external loops (EL): EL1 (Figure 1A, pink) and EL5 (Figure 1A, gray). The N-terminus of EL5 is unstructured when only the acceptor substrate is bound (Lizak et al., 2011) and becomes ordered upon binding of LLO (Napiórkowska et al., 2017). The periplasmic domain (residues 433–711; Figure 1A, green) exhibits a mixed α/β fold with two major structural elements: the insertion domain and the conserved central core (Maenaka et al., 2008; Maita et al., 2010). The insertion domain is the beta-barrel-like structure of the periplasmic domain and its function is currently unknown; the central core contains residues important for recognition and binding of the acceptor substrate (Maita et al., 2010). The acceptor substrate is bound in a large cavity at the interface between the transmembrane and periplasmic domains and is pinned by the structured C-terminus of external loop EL5 (Lizak et al., 2011) (Figure 1A, red). LLO binds in the second large cavity located opposite the acceptor substrate binding cavity; the two cavities are linked by the acceptor asparagine (Napiórkowska et al., 2017). The crystal structures suggest a glycosylation mechanism where the catalytically essential and highly conserved magnesium coordinating residues D56 (EL1) and E319 (EL5) form hydrogen bonds with the acceptor asparagine and prime it for nucleophilic attack on LLO (Lizak et al., 2011; Napiórkowska et al., 2017) (Figure 1B and Supplementary Figures S1A,B, conserved residues in red).

While the typical consensus sequence of OSTs consists of N-X-S/T (X ≠ P), a threonine residue at the +2 position (where the acceptor asparagine is defined as position 0) allows for glycosylation 40 times more efficiently in eukaryotes than a serine in this position (Bause, 1984). In *C. lari* PglB, N-X-S peptides have been reported to have 4-fold reduced peptide binding affinity and turnover is 1.2-fold faster for N-X-T sequences (Gerber et al., 2013). In the crystal structure of PglB, a threonine at position p+2 of the peptide forms hydrogen bonds with the strictly conserved WWD motif (residues 463–465; Supplementary Figure S1C, red) and a van der Waals contact with I572 (3.6 Å distance between gamma-methyl groups) (Lizak et al., 2011). One obvious explanation for the difference in catalytic activity between N-X-S and N-X-T is that serine cannot form a van der Waals contact with I572; supporting this is that an I572 V mutant with N-X-T peptides had a similar 4-fold reduction in peptide binding affinity (Gerber et al., 2013). However, the lost van der Waals interaction is a small contribution compared to the multiple hydrogen bonds with the WWD motif (which are not predicted to be affected), making it difficult to explain the significant effect on glycosylation. For this reason, we performed molecular dynamics simulations to understand the change in substrate-enzyme interactions between the two amino acids at position p+2 of the substrate.

In this paper we focus on the substrate binding phase of the catalytic cycle and assess the differences that could affect the catalytic competency of PglB when bound to acceptor substrates containing threonine or serine at the +2 position using molecular dynamics (MD) simulations. The catalytic activity of PglB requires binding of both the acceptor substrate and LLO, although the order is thought to be unimportant (Lizak et al., 2011; Napiórkowska et al., 2017). While LLO may bind before the acceptor substrate, to our knowledge there is no evidence that LLO binding is the rate limiting step. Thus, understanding PglB-substrate interactions and the propensity of the complex to form a catalytically competent state suitable for LLO attachment can shed light on the observed differences in catalytic efficiency.

MD simulations of *C. lari* PglB with the optimal acceptor sequence, GDQNATFG (PglB-NAT), and the peptide variant GDQNASFG (PglB-NAS) were used to discover the detailed differences between threonine and serine at the +2 position in the substrate-bound state of the catalytic cycle. We observed an increased propensity for substrate disassociation in the trajectories of PglB-NAS compared to PglB-NAT. Our results show that threonine at the +2 position forms a network of interactions with the periplasmic domain and EL5 that promotes a more closed orientation of the periplasmic domain. The loss of the methyl group in the serine disrupts the substrate-enzyme interaction and the allosteric communication between the periplasmic domain and external loop EL5, allowing for increased motion of the periplasmic domain and leading to a smaller population of stably bound, catalytically competent states.

## Materials and Methods

### Simulation Protocol

Molecular dynamics simulations of the bacterial OST PglB from *C. lari* with bound optimal peptide (GDQNATFG) were started from the crystal structure (PDB code 3RCE). The missing residues of external loop EL5 (residues 283–306) and missing loop residues in the periplasmic domain (residues 605–607) were built with modeler (Fiser et al., 2000) using a protocol that optimizes the positions of all non-hydrogen atoms of the loop without altering the coordinates of the existing residues. The optimization protocol used conjugate gradient minimization followed by molecular dynamics with simulated annealing (Fiser et al., 2000). The missing residues of the EL5 loop (residues 283–306) remain mostly disordered during the simulations, but they sample α-helical conformations with a probability of ∼5%. Residues 284–289 are known to become structured and form a α-helix upon binding LLO (Napiórkowska et al., 2017). Using VMD (Humphrey et al., 1996), hydrogens were added to the crystal structure, the protein and peptide were inserted into a lipid bilayer, and the system was solvated with TIP3P (Jorgensen et al., 1983; MacKerell et al., 1998) water (the PglB/peptide system was electrostatically neutral). To evaluate the effect of the lipid content of the bilayer on the dynamics of PglB, we used either POPC or POPE lipid bilayers.

The simulations were performed with NAMD 2.13 (Phillips et al., 2005) using the CHARMM27 force field for proteins and lipids (MacKerell et al., 1998). In the first phase of the equilibration, all atoms except the hydrocarbon tails of the POPC or POPE lipids were fixed. Conjugate gradient minimization followed by equilibration with constant temperature and volume (NVT ensemble) allowed the lipid tails to melt while the water, lipid head groups, Mg^2+^ ions, PglB, and peptide positions remained fixed. The following steps were then performed thirteen times to generate independent trajectories (ten with POPC lipids, three with POPE lipids). In the second phase of equilibration with constant temperature and pressure (NPT ensemble), restraints were removed and a force was applied to any water molecules that entered the lipid bilayer to prevent infiltration and disruption of the bilayer. At the beginning of the second phase, conjugate gradient minimization was performed on all atoms, followed by velocity reinitialization and temperature and pressure equilibration. The final phase of equilibration was performed with no constraints or applied forces using constant temperature and pressure to allow for final density equilibration of the system. Multiple restraint conditions were tried in the final phase of equilibration and the unrestrained equilibration protocol was determined to be the most appropriate to reproduce the coordination of the Mg^2+^ ion given the uncertainty in the Mg^2+^ coordination due to resolution of the crystal structure (3.4 Å) (Lizak et al., 2011). The coordination obtained by unrestrained equilibration is consistent with previous molecular dynamics simulations of PglB (Pedebos et al., 2015; Lee and Im, 2017).

The production phase of the data generation used constant temperature, pressure, and constant area in the plane of the bilayer (NP_xy_P_z_T ensemble). Pressure and temperature were maintained at 1 atm and 310 K, respectively, using Langevin dynamics and the Nosé-Hoover Langevin piston method. The SHAKE constraint algorithm (Ryckaert et al., 1997) was used to allow a 2 fs time step and the particle mesh Ewald method (Essmann et al., 1995) was used for electrostatic interactions with periodic boundary conditions. Thirteen independent trajectories (ten with POPC lipids, three with POPE lipids) of 500 ns each were collected for PglB with the GDQNATFG peptide (PglB-NAT). The consensus sequence variant (T→S) was created with the Mutator plugin of VMD (Humphrey et al., 1996) and thirteen independent trajectories of up to 500 ns each (ten with POPC lipids, three with POPE lipids) of PglB with the GDQNASFG peptide (PglB-NAS) were collected using the same simulation protocol as above. Three of the trajectories (1, 5, and 12) were terminated after observing peptide unbinding (at 400 ns, 100 and 300 ns, respectively, see *Definition of Peptide Binding*).

The analysis presented in this paper focuses on differences in the bound states of the two peptides. The results of the simulations that used POPC and POPE lipids were found to be the same within the error of the measured observables (interaction energies, H-bond probabilities, distances between PglB and key peptide residues, etc.), indicating that the dynamics of PglB is not influenced by the lipid composition of the bilayer. For example, the difference in the peptide-PglB interaction energy for the two lipid types was less than the standard deviation: PglB-NAT POPC = −228.7 ± 24.8 kcal/mol, PglB-NAT POPE = −240.0 ± 26.7 kcal/mol, Δ(NAT POPC-POPE) = 11.3 kcal/mol. Therefore, we combined all the trajectories for better sampling. PglB-NAT spent 5,063 ns in the bound state and PglB-NAS spent 2,780 ns in the bound state and all histograms were normalized to account for the difference in the number of data points. To ensure that we have collected enough of the relevant states to be able to make meaningful comparisons between the two peptides, we assessed the quality of sampling using jack-knife analysis (Lohr, 1999) of the peptide-PglB interaction energy. The standard deviation of the jack-knife means was calculated and found to be approximately 1/10 of the standard deviation of the interaction energy, indicating that we have good sampling (Supplementary Table S1). Furthermore, a jack-knife style analysis of the first two principal components (where each trajectory is excluded in turn) of the bound frames shows that the trajectories sample similar regions of conformational space independent of the lipid type (POPC: trajectories 1–10, POPE: trajectories 11–13), another indicator of adequate sampling (see Supplementary Figures S2, S3).

### Analysis of Trajectories

Except where otherwise specified, trajectory analysis was performed with VMD (Humphrey et al., 1996) on bound frames only (see *Definition of Peptide Binding*) excluding the first 50 ns to allow for structural equilibration, figures were prepared with Tableau Software v10.4 (Seattle, WA), and structures were visualized with VMD using the STRIDE algorithm for secondary structure identification (Frishman and Argos, 1995).

#### Hydrogen Bonds

A hydrogen bond is defined by a donor-acceptor distance of less than 3.5 Å and a donor-hydrogen-acceptor angle of 130° < θ < 180°.

#### Definition of Peptide Binding

During the simulations we observed that the peptide could be in different states: bound, partially bound, or unbound from the enzyme. We identified six non-catalytic H-bonds between the enzyme and the peptide as important indicators of peptide binding (see Table 1). The presence or absence of these H-bonds are used as the criteria for the bound, partially bound, and unbound states (Table 2). In addition, trajectories were terminated when the peptide had moved far away from the binding pocket of the enzyme. This was quantified using the sum of the donor-acceptor distances of three H-bonds formed by the peptide with the binding pocket (p+1 O-M318 N, p+2 Oγ-W463 Nε, and p+2 N-D465 Oδ) plus the distance between p+2 Cβ-I572 Cδ (a proxy for the p+2-I572 van der Waals interaction). Trajectories were terminated when this sum of distances exceeded 80 Å (see Supplementary Figures S4, S5). In the bound state, the average of this sum is 13.9 ± 1.8 Å for PglB-NAT and 17.8 ± 4.4 Å for PglB-NAS (the average individual distances are listed in Supplementary Table S2).

**TABLE 1:** Probability of the two peptides (NAT and NAS) forming the H-bonds identified as important in binding and the catalytically relevant H-bonds (mean and standard deviation for bound states).

	p (PglB-NAT)	p (PglB-NAS)
p+1 O-M318 N	0.83 ± 0.24	0.61 ± 0.35
p+2 N-D465 Oδ^a^	0.91 ± 0.10	0.68 ± 0.20
p+2 Oγ-W463 Nε	0.78 ± 0.20	0.38 ± 0.27
p+2 Oγ-W464 Nε	0.32 ± 0.17	0.16 ± 0.10
p+2 Oγ-D465 Oδ^a^	0.92 ± 0.11	0.58 ± 0.19
p+3 N-T316 O	0.81 ± 0.31	0.36 ± 0.31
p0 Nδ-D56 Oδ^a^	0.35 ± 0.35	0.41 ± 0.28
p0 Nδ-E319 Oε^a^	0.25 ± 0.27	0.21 ± 0.25

a Combined probability of hydrogen bond formation with either Oδ_1_/Oδ_2_ or Oε_1_/Oε_2_.

**TABLE 2:** Definition of peptide state relative to the PglB binding pocket, based on peptide-PglB interactions.

State	Criteria
Fully bound	Presence of at least one of the following H-bonds: p+1 O-M318 N
	p+2 Oγ-W463 Nε
	p+2 N-D465 Oδ
	AND presence of at least one of the following H-bonds: p+2 Oγ-W464 Nε
	p+2 Oγ-D465 Oδ
	p+3 N-T316 O
Partially bound	All the following H-bonds are lost for >5 ns: p+1 O-M318 N
	p+2 Oγ-W463 Nε
	p+2 N-D465 Oδ
	AND presence of at least one of the following H-bonds: p+2 Oγ-W464 Nε
	p+2 Oγ-D465 Oδ
	p+3 N-T316 O
Unbound	All the following H-bonds are lost for >5 ns: p+1 O-M318 N
	p+2 Oγ-W463 Nε
	p+2 N-D465 Oδ
	p+2 Oγ-W464 Nε
	p+2 Oγ-D465 Oδ
	p+3 N-T316 O
Trajectory termination	After the sum of the distances (p+1 O-M318 N) + (p+2 Oγ-W463 Nε) + (p+2 N-D465 Oδ) + (p+2 Cβ-I572 Cδ) exceeds 80 Å at any point in the trajectory

#### Average and Most Populated Structures

The average bound structure was generated by averaging over all bound frames for each system. The average structure representing the unbound state was generated by averaging over all frames of PglB-NAS after the loss of the six non-catalytic H-bonds identified as important indicators of binding (see Table 1). To validate the use of average structures, the Ramachandran plot of the average structures was compared to the ProCheck (Laskowski et al., 1993) Ramachandran plot for the crystal structure. Only residues in solvent exposed turn/coil regions of the average structures were found to be outside of the allowed area predicted for the crystal structure: E258, G281, K296, and L303. E258 is also outside of the Ramachandran plot in the crystal structure. G281, K296, and L303 are in the highly flexible unstructured EL5 N-terminal region absent from the crystal structure.

To identify the most populated structure we used two parameters: 1) the largest difference in the Cα-Cα distance between the periplasmic and transmembrane domains (residues 535–537 of the periplasmic domain and residue 279 of the transmembrane domain) and 2) the backbone RMSD (root mean square displacement). From the trajectories of PglB-NAT and PglB-NAS we identified the structures corresponding to the most populated inter-domain distance (PglB-NAT: 44.1 Å, PglB-NAS: 48.4 Å) and RMSD values. These structures were then validated as representative of the most populated conformation by locating them in the plot of the first two principal components and were found to be located in the regions of highest density (Supplementary Figure S6).

RMSD between average or most populated structures was calculated as

$$RMSD\left(NAT_{ave}-NAS_{ave}\right)=\sqrt{{\left(\frac{1}{T}\overset{T}{\underset{t}{\sum }}NAT-\frac{1}{T}\overset{T}{\underset{t}{\sum }}NAS\right)}^{2}},$$ (1)

where NAT and NAS are the subset of backbone atoms of PglB-NAT and PglB-NAS, respectively, for which the RMSD is calculated.

#### Structural Alignment (Identification of the Stable Core)

The choice of residues to use for structural alignment is important in cases where domain motion occurs. A poor choice of alignment can obscure differences while a good choice will highlight them. Alignment on the transmembrane domain (excluding EL1 and EL5) was found to perform better than an all-atom alignment. However, previous simulations of PglB have found that the transmembrane domain does not behave as a single rigid domain (Lee and Im, 2017). To further refine the choice of alignment residues, the trajectories were analyzed with the *bio3d* geostas algorithm (Grant et al., 2006; Romanowska et al., 2012) in the R environment (R development core team, 2006; http://www.R-project.org) to determine which residues have the least variation in position over the trajectory (the stable core). The stable core residues were determined from the combined trajectories of PglB-NAT and PglB-NAS and consist of transmembrane domain residues 118–124, 126–130, 132, 342–347. Alignments on all atoms of PglB, the transmembrane domain, and the stable core were compared for structural analysis, cross-correlation calculations, and principal component analysis. In all cases, alignment on the stable core identified by the geostas algorithm (Romanowska et al., 2012) performed the best at highlighting relevant differences between the systems. The stable core was used for structural alignment to remove rotational and translational motion in all analyses reported here.

#### Principal Component Analysis

Cartesian principal component analysis was performed for bound frames only excluding the unstructured N-terminus of EL5 (residues 282–306) using the *bio3d* package (Grant et al., 2006) in the R environment (R development core team, 2006; http://www.R-project.org). The structured C-terminus of EL5 (residues 307–324), which is present in the crystal structure (Lizak et al., 2011), is included in the calculations. The calculation used a single combined trajectory for both systems aligned on the stable core identified by the *bio3d* geostas algorithm (Romanowska et al., 2012). Initial analysis of the trajectories showed a high similarity in the principal components of PglB-NAT and PglB-NAS (with a root mean square inner product (Grant et al., 2006) of the principal components of 0.84); because of the similarity in the principal components, the trajectories were combined in the final analysis to facilitate the comparison of the conformational space sampled by PglB-NAT and PglB-NAS. The first two principal components (PC1 and PC2) are representative of the dynamics of the combined system, capturing 84 and 9% of the motion, respectively. Density heat maps of the first two principal components were generated using the *mclust* R package (Fraley and Raftery, 2002).

#### Interaction Energy

The interaction energy was calculated using the namdenergy plugin of VMD (Humphrey et al., 1996). For PglB/p+2 interaction energies by residue, backbone and sidechain distances were used to determine which residues of PglB were close enough to interact for computational efficiency. The interaction energy for all residues with a center of mass distance within 15 Å of p+2 was found to be in excellent agreement with the total p+2/PglB interaction energy (see Supplementary Table S3). The interaction energy between the peptide and the catalytic Mg^2+^ ion was also calculated and the results did not alter the conclusions based on the PglB/peptide interaction energy (Supplementary Table S4).

#### Network Analysis

Network analysis was performed by the *bio3d* package (Grant et al., 2006) in the R environment (R development core team, 2006; http://www.R-project.org) using cross-correlations calculated over all bound frames aligned on the stable core. Cross-correlations between PglB, the peptide, and Mg^2+^ ions were included in the calculations with a threshold of 0.7 (corresponding to an angle between motion vectors of <45°). Community membership was determined using the greedy algorithm (Clauset et al., 2004) and simplified network graphs were pruned of unconnected communities.

Network graphs overlaid on the structure represent each community by a sphere located at the geometric center of each community. The community radius (R_i_) and edge weight between two communities (E_ij_) are defined as

$$R_{i}=\frac{\sqrt{N_{i}}}{3}$$ (2)

$$E_{ij}=\frac{-ln\left|\text{max}\left(C_{ij}\right)\right|}{4},$$ (3)

where N_i_ is the number of residues in each community and max (C_ij_) is the maximum correlation coefficient between residues in each connected pair of communities. The scaling was chosen to illustrate relative differences between communities without obscuring the underlying structure. Residues of the structure are colored according to community membership with unconnected communities in white in Supplementary Figure S7A. Simplified network graphs generated in R (Supplementary Figures S7B,C) follow the same community coloring (unconnected communities not shown) with community radii and edge weights as defined below.

$$R_{i}=\frac{N_{i}}{3}$$ (4)

$$E_{ij}=-ln\left|max\left(C_{ij}\right)\right|.$$ (5)

The number of shortest paths that pass through each node (betweenness), the number of edges of each node (degree), and suboptimal path analysis are calculated from the full network defined by the underlying cross-correlation matrix (where each residue is a node). The nodes contained in the suboptimal paths of PglB-NAT were identified using 500 paths and visualized using the first 100 paths for clarity.

#### Sequence Alignment

Sequence alignments were performed using with the Clustal algorithm (Madeira et al., 2019) on the reviewed sequences of InterPro 83.0 (December 2, 2020), family IPR003674 (29 sequences) (Blum et al., 2021). A subset of these sequences was chosen to highlight conservation across the three families (prokaryotes, archaea, and eukaryotes, Supplementary Figures S1A–C) or the presence of a longer hydrophobic motif after the DXNK motif among eukaryotes (Supplementary Figure S1D).

## Results

### Differences in the Bound State of NAT and NAS Peptides

Molecular dynamics simulations of *C. lari* PglB with the optimal acceptor sequence, GDQNATFG (PglB-NAT), and the peptide variant GDQNASFG (PglB-NAS) were used to discover the detailed differences between threonine and serine at the +2 position in the substrate-bound state of the catalytic cycle. Thirteen replicas with a cumulative simulation time of 6.5 and 5.8 μs were collected for PglB-NAT and PglB-NAS, respectively. We observed a striking difference in the behavior of NAT and NAS peptides: out of the thirteen independent trajectories collected for each system, four trajectories of PglB-NAT exhibit partial peptide unbinding while ten trajectories of PglB-NAS exhibit partial peptide unbinding. Since a stably bound substrate is prerequisite to forming a catalytically competent state, we wanted to understand the critical interactions in the bound state that lead to this behavior. To do so, we needed to quantitatively determine when the peptide was in the bound state versus a partially bound state or fully unbound state in our simulations. We then analyzed the frames where the peptide is bound to identify differences in the bound states of the two peptides.

We identified six non-catalytic H-bonds whose absence corresponds to loss of the catalytically relevant H-bonds and disassociation from the binding pocket. These H-bonds were used to define the bound, partially bound, and fully unbound states (see Table 2). The H-bonds p+1 O-M318 N, p+2 N-D465 Oδ, and p+2 Oγ-W463 Nε (see Supplementary Figures S4, S5 for donor-acceptor distances) provide a good indicator of partial unbinding because once all three are lost, with no specific order, the peptide no longer simultaneously forms the catalytically relevant H-bonds with D56 and E319 (the definition of the partially bound state as used in this work) and stable rebinding is never observed.

**FIGURE 2:**
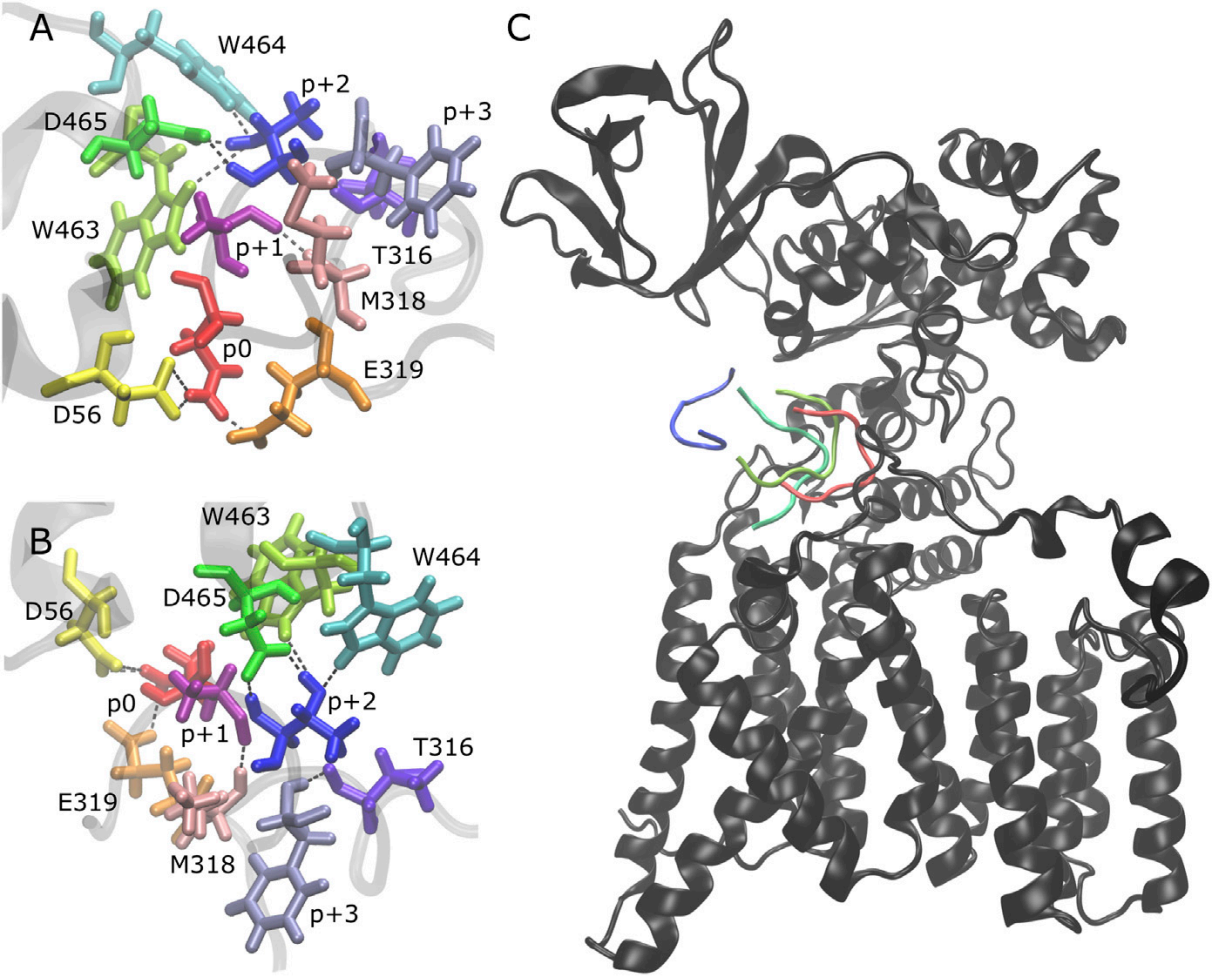
Peptide unbinding and key hydrogen bonds. **(A,B)** Front and side view of key hydrogen bonding pairs between peptide and PglB (NAT peptide shown, backbone of peptide and adjacent PglB residues in transparent gray): catalytically relevant H-bond pairs between p0 (red) and D56 (yellow)/E319 (orange); backbone H-bond pairs of EL5 C-terminus residues T316 (violet) and M318 (light pink) with p+3 (lavender) and p+1 (dark pink), respectively; H-bond pairs between p+2 (blue) and periplasmic domain residues W463 (lime), W464 (cyan), and D465 (green). **(C)** Example of peptide unbinding from PglB-NAS, trajectory 1. PglB (black) is shown in the fully bound conformation after equilibration (*t* = 50 ns). The NAS peptide is shown at different time points in the unbinding process: fully bound after equilibration (red, *t* = 50 ns) and during unbinding (light green, *t* = 276 ns; dark green, *t* = 291 ns; blue, *t* = 307 ns).

We analyzed the behavior of the peptides after becoming partially bound and observed that there is no clear pathway that leads to the full unbinding and dissociation from the binding pocket. After the loss of the H-bonds p+1 O-M318 N (Figures 2A,B, Supplementary Figure S8: dark pink/light pink), p+2 N-D465 Oδ (Figures 2A,B, Supplementary Figure S8: blue/green), and p+2 Oγ-W463 Nε (Figures 2A,B, Supplementary Figure S8: blue/lime), the peptide is still in the binding pocket but is more loosely associated with the enzyme (Figure 2C, light green). The peptide can remain in this state for the remainder of the simulation time or it can become fully disassociated (for example, Supplementary Figure S5: trajectory 3 vs. trajectory 1). The presence of three additional H-bonds keeps the peptide in this partially bound state: p+2 Oγ-W464 Nε (Figures 2A,B, Supplementary Figure S8: blue/cyan), p+2 Oγ-D465 Oδ (Figures 2A,B, Supplementary Figure S8: blue/green), and p+3 N-T316 O (Figures 2A,B, Supplementary Figure S8: violet/lavender). Upon losing these H-bonds (which are never observed to reform once all three are lost), the peptide moves further away and becomes fully dissociated from the binding pocket (Figure 2C, dark green and blue). Thus, our results suggest that multiple interactions are important for the stability of the bound substrate: three key H-bonds are essential to keep the peptide tightly bound in the binding pocket of the enzyme (p+1 O-M318 N, p+2 N-D465 Oδ, and p+2 Oγ-W463 Nε) and three additional H-bonds keep the peptide in a partially bound state and prevent the full dissociation from the binding pocket (p+2 Oγ-W464 Nε, p+2 Oγ-D465 Oδ, and p+3 N-T316 O).

**FIGURE 3:**
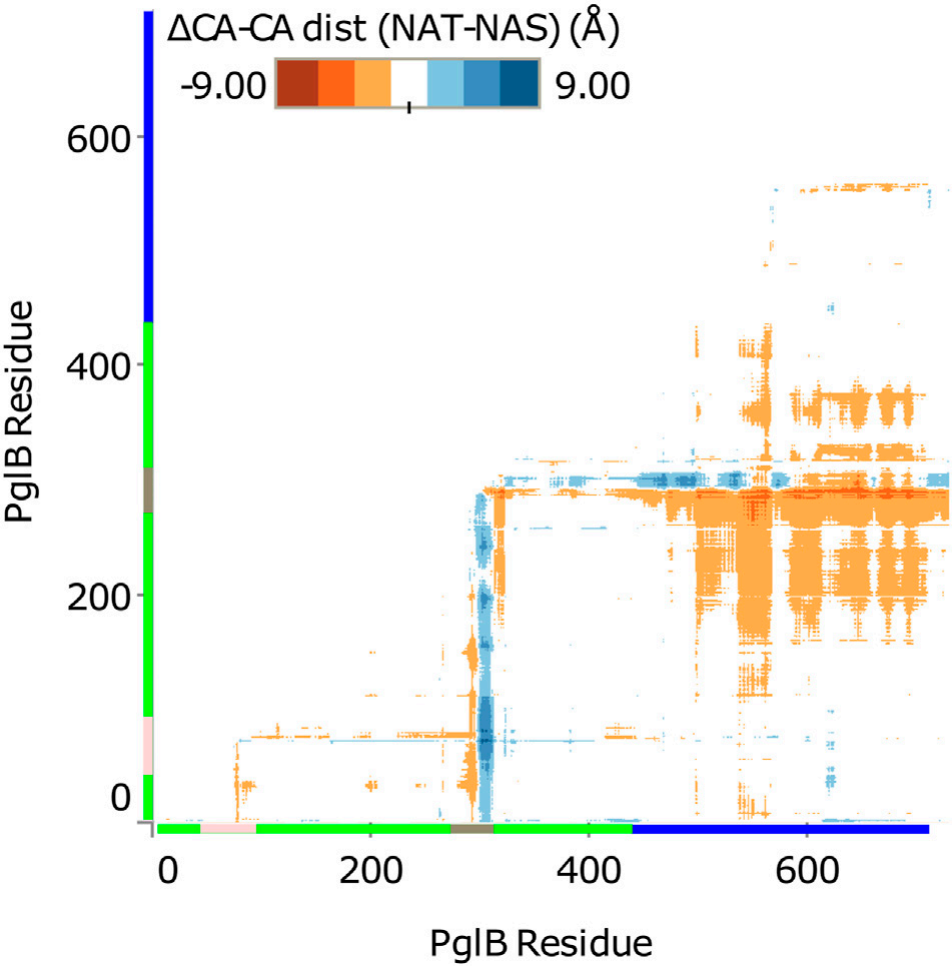
Difference in Cα-Cα distance between PglB-NAT and PglB-NAS. Colored axis bars mark the different domains. Green: transmembrane domain, pink: external loop EL1, gray: external loop EL5, blue: periplasmic domain.

The probabilities of all six of the hydrogen bonds that stabilize the bound and partially bound states are decreased in PglB-NAS compared to PglB-NAT (Table 1). To evaluate the stability of the bound substrate peptides, we calculated the probability of maintaining or transiently losing the first three and all six of these bonds simultaneously. We find that the NAS peptide is much less stably bound than the NAT peptide. All three of the H-bonds whose presence defines the bound state are simultaneously maintained in 68% of PglB-NAT bound states while only 24% of PglB-NAS bound states meet this criterion. Similarly, the probability of maintaining all six of these H-bonds is decreased and the probability of transiently losing all three or six of these H-bonds is increased by factors of 5–10 for PglB-NAS (Supplementary Table S5). This difference is seen over all trajectories and when comparing only those trajectories where the peptide eventually becomes partially bound (Supplementary Table S5, starred probabilities). Surprisingly, when comparing the peptides in the bound state, they have the same probability of forming the catalytically relevant H-bonds p0 Nδ-D56 Oδ (Figures 3A,B, Supplementary Figure S8: red/yellow) and p0 Nδ-E319 Oε (Figures 3A,B, Supplementary Figure S8: red/orange) (Table 1), which are formed transiently in the absence of LLO [as seen previously (Pedebos et al., 2015)]. However, when considering the full trajectories (bound, partially bound, and unbound frames) of PglB-NAT and PglB-NAS, the population of catalytically competent states (where both catalytically relevant H-bonds are present) is lower for the NAS peptides by half compared to NAT peptides (Supplementary Table S6) because the NAS peptides populate the bound state with lower probability (Supplementary Table S6). From this we conclude that the differences between NAT and NAS peptides that lead to increased unbinding in NAS are crucial to understanding the difference in catalytic competency.

None of the six H-bonds identified as important for stable binding should be impacted by the T/S variation in the consensus sequence at position p+2 and two out of six (p+1-M318 and p+3-T316) do not involve p+2 at all. To understand the difference in stability of the bound NAT and NAS peptides, we calculated the interaction energy between the peptide residues and PglB. The largest change is observed for p+2, however, the interaction energy while bound is affected beyond this residue and shows a large degree of compensation at peptide residue p-2 (Table 3).

**TABLE 3:** Difference in PglB-peptide interaction energy between PglB-NAT and PglB-NAS by peptide residue.

Peptide residue	ΔPglB Int. Energy (NAT-NAS) (kcal/mol)
p-3 (Gly)	−3.1
p-2 (Asp)	10.5
p-1 (Gln)	2.0
p0 (Asn)	2.4
p+1 (Ala)	−3.0
p+2 (Thr/Ser)	−11.1
p+3 (Phe)	−2.7
p+4 (Gly)	−1.8
Total	−6.8

The interaction energy between PglB and the NAS peptide is overall less favorable (Table 3), consistent with increased disassociation. To understand the changes in conformation underlying the altered interaction energy, we compared the average bound structures of PglB-NAT and PglB-NAS and found differences in both the conformations of the peptide and of the periplasmic domain. The backbone RMSD between average structures (see Materials and methods, Eq. 1) is 1.46 Å for the peptide and 2.48 Å for the periplasmic domain. In contrast, the transmembrane domain shows minimal change between the two structures (backbone RMSD of 0.73 Å).

The compensation observed in the interactions with p-2 can be explained by the changes observed in the NAS peptide. The aspartic acid at position p-2 shifts relative to its transmembrane domain hydrogen-bonding partners R147 and R331, decreasing the favorable interaction energy with R147 (PglB-NAT interaction more favorable by −4.1 kcal/mol) and increasing the favorable interaction energy with R331 (PglB-NAS interaction more favorable by −12.6 kcal/mol), accounting for 80% of the difference in interaction energy at p-2. The remaining difference comes from numerous long-range electrostatic interactions of the charged aspartic acid side chain.

For p+2, the key interacting residues of the WWD motif and I572 from the crystal structure (Lizak et al., 2011) show deviations in average position between PglB-NAS and PglB-NAT. The backbone RMSD between average structures is 0.87–1.18 Å for the WWD motif, 1.67 Å for I572, and 0.93 Å for p+2, indicating that conformational change of both the peptide and the periplasmic domain contribute to the differences in the interaction energy.

### The Substrate Influences the Periplasmic Domain Orientation of PglB

Comparison of the average structures suggests a difference in the conformation of PglB when bound to NAT versus NAS peptides. The difference between PglB Cα-Cα distances was calculated for PglB-NAT and PglB-NAS to identify regions of PglB that differ in conformation when bound to the two peptides. We found that the distance between the periplasmic domain and the transmembrane domain is increased in PglB-NAS compared to PglB-NAT (Figure 3). The average Cα-Cα distance between the periplasmic domain and the transmembrane domain (excluding the large external loops EL1 and EL5) is increased by 0.8 Å per residue pair in PglB-NAS compared to PglB-NAT, indicating that on average, the region between the two domains (containing the catalytic pocket) is more open in PglB-NAS (designated the open state) than in PglB-NAT (designated the closed state).

In contrast, the intradomain Cα-Cα distances of the periplasmic domain and the transmembrane domain differ remarkably little between PglB-NAT and PglB-NAS, with average changes of 0.01 Å and 0.04 Å per residue pair, respectively. The minimal change in Cα-Cα distances within the periplasmic and transmembrane domains indicates that the overall structure of both the periplasmic and transmembrane domains do not change between PglB-NAS and PglB-NAT. These results suggest that while the structure of the two domains is conserved, their relative positions change and that there should be a “hinge” that allows for rigid domain motion. If true, we would expect the hinge to meet the following criteria: 1) the hinge should have low backbone RMSD between average structures, 2) the hinge should be located in an interfacial region between transmembrane and periplasmic domains, and 3) the magnitude of the displacement observed between the open and closed positions should correlate with the distance from the hinge. In our system, this means we expect that the further a residue is from the hinge, the more different its position will be between the open state and the closed state for the mobile portion of the structure (the periplasmic domain). Two pairs of structures were used to identify the differences between PglB-NAS and PglB-NAT: the average structure and as an additional validation, the most populated structure (calculated as described in *Analysis of Trajectories*). The results obtained using either the average or the most populated structure are the same.

**FIGURE 4:**
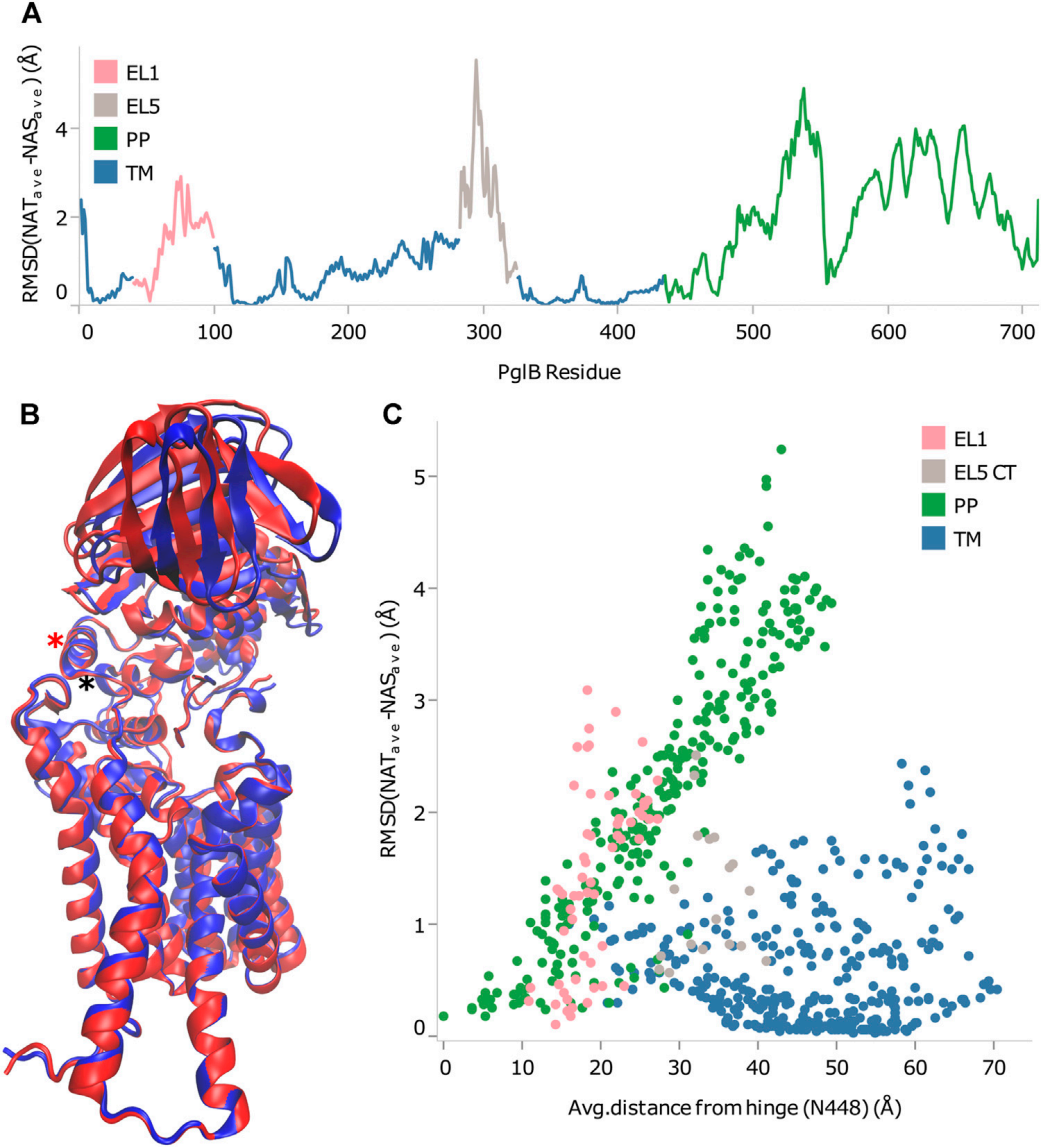
Identification of rigid-body periplasmic domain motion hinge. **(A)** Backbone RMSD by residue between average bound structures of PglB-NAT and PglB-NAS colored by domain. Blue: transmembrane domain (TM), pink: external loop EL1, gray: external loop EL5, green: periplasmic domain (PP). **(B)** Average bound structure of PglB-NAT (blue) and PglB-NAS (red) aligned on consensus stable core residues (unstructured portion of EL5 not shown). Red asterisk marks helix containing N448, black asterisk marks end of WWD helix at Y473. **(C)** Distance from N448 in the average bound structure of PglB-NAT versus backbone RMSD between average bound structures of PglB-NAT and PglB-NAS for each residue of PglB, colored by domain. Blue: transmembrane domain (TM), pink: EL1, gray: EL5, green: periplasmic domain (PP).

From the RMSD between of PglB-NAT and PglB-NAS, we chose residues with low RMSD in regions of the structure appropriate for the hinge. We then calculated the correlation of the backbone RMSD and the distance from each of the chosen residues to see which of these residues agreed best with the criteria for the hinge residue (Supplementary Table S7). From this, we found that N448 and Y473 best meet the criteria for the hinge between the closed state (PglB-NAT) and the open state (PglB-NAS). Both residues are located in regions of low RMSD (Figure 4A) and have the highest correlation coefficient (0.95) between distance and backbone RMSD of the periplasmic domain residues (Supplementary Table S7). These two residues are adjacent in the structure of PglB at the interface between the two domains (Figure 4B): N448 (red asterisk) is located on a helix in between the transmembrane domain and the periplasmic domain with Y473 directly below N448 at the terminus of the helix containing the WWD motif (black asterisk), indicating that the hinge is located at the junction of these two helices. Additionally, the backbone RMSD between the periplasmic domain residues (the mobile portion of the structure) increases linearly with distance from N448 and Y473 but the correlation is weak for other regions of PglB (see Figure 4C for example). Lastly, we chose other residues distant from the hinge region with low RMSD across the sequence of PglB and these residues show low correlation between distance and RMSD (Supplementary Table S8), validating that the strong linear relationship is unique to the hinge region.

### Origin of Observed Structural Differences

**FIGURE 5:**
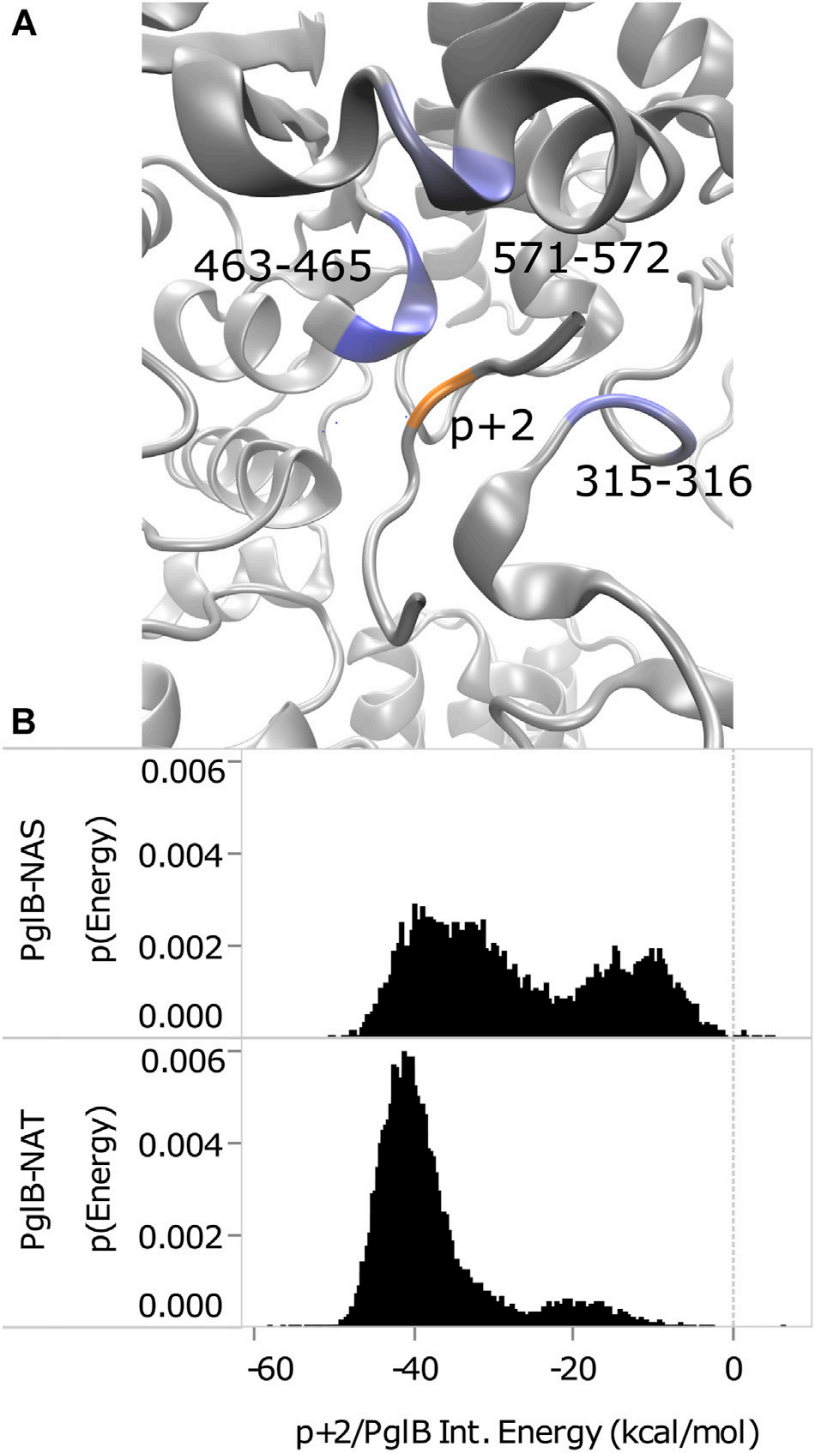
Comparison of p+2/PglB interaction energy for PglB-NAT and PglB-NAS. **(A)** Differences in interaction energy between PglB and p+2 (NAT-NAS, blue = more favorable in PglB-NAT). Backbone of peptide residue p+2 shown in orange. **(B)** Comparison of total p+2/PglB interaction energy for PglB-NAT and PglB-NAS.

The interaction energy between p+2 and the residues of PglB was calculated for both peptides (Table 4) to understand how the T/S difference results in widespread changes in the peptide-PglB interaction (Table 3) and how it affects the orientation of the periplasmic domain (Figure 3). The location of residues that differ in interaction energy between PglB-NAT and PglB-NAS by ≥ |±0.5| kcal/mol (absolute value of the difference ≥0.5) are shown in Figure 5A. For each of these residues, the interaction energy is less favorable in PglB-NAS (Table 4). As expected, the loss of the methyl group in p+2:S decreases the interaction energy with I572 but also its neighbor R571. The WWD motif (463–465) shows the largest differences in interaction energy and surprisingly, residues in the structured C-terminal portion of EL5 (315–316) show significant differences as well.

**TABLE 4:** Interaction energy between p+2:T and residues of PglB and the difference between p+2:T and p+2:S interaction energies for residues where the absolute value of the difference in interaction energy is ≥0.5 kcal/mol.

PglB residue	p+2/PglB energy (NAT) (kcal/mol)	Δp+2/PglB energy (NAT-NAS) (kcal/mol)
E315	−0.9 ± 0.4	−0.6
T316	−3.4 ± 1.3	−1.2
W463	−3.0 ± 1.4	−2.0
W464	−3.0 ± 1.5	−1.3
D465	−20.7 ± 5.7	−4.4
R571	−1.5 ± 0.9	−0.5
I572	−1.8 ± 0.5	−1.0
Total	−34.4 ± 6.9	−11.0

Although the average differs by ≥ |±0.5| kcal/mol for the residues listed in Table 4, a histogram of the interaction energy shows that the two peptides sample states with similar p+2 interaction energies (Supplementary Figure S9). The measured difference in the average interaction energy of p+2 is due to the higher population of states with near-zero interaction energies by the NAS peptide (Supplementary Figure S9), decreasing the average interaction energy of the residues highlighted in Figure 5A. This suggests that p+2:S interacts in a similar way with residues of PglB where the interaction energy differs by ≥ |±0.5| kcal/mol, but that the interactions are less stable, consistent with the decreased stability of H-bonds identified as important for peptide binding involving many of these same residues (Table 1 and Supplementary Table S5).

The total effect of sampling higher energy conformations with higher probability in PglB-NAS can be seen in the total interaction energy between p+2 and PglB (Figure 5B). Both PglB-NAT and PglB-NAS have bimodal distributions indicating the sampling of two states, one more favorable than the other. However, the more favorable (primary) mode and the less favorable (secondary) mode are more favorable in PglB-NAT by approximately 6 and 7 kcal/mol, respectively. Additionally, the more favorable state is more populated in both PglB-NAT and PglB-NAS but the difference in the population of the two states is significantly larger for PglB-NAT (Figure 5B).

These data indicate that the loss of the methyl group in p+2:S results in fewer interactions between the peptide and PglB. The result of these changes is less favorable total interaction energy in PglB-NAS (Figure 5B) due to fewer simultaneous interaction partners. The interactions of the residue at p+2 with the periplasmic domain and the EL5 loop provide a mechanism for the T/S variation at the +2 position to affect the periplasmic domain orientation: threonine may act as a pin to close a latch, preventing opening of the periplasmic domain hinge (near N448/Y473). We define residues of PglB with an absolute difference in the p+2 interaction energy ≥1.0 kcal/mol as the latch residues (W463 and D465 of the WWD motif, I572, and EL5 residue T316). Our hypothesis is that p+2 interacting with these latch residues acts as a pin to close the latch which limits the hinge motion when p+2 is threonine, but that this ability is impaired when p+2 is serine. The two sides of the latch are formed by the interacting residues of the periplasmic and EL5 domains (Figure 5A) and by linking the two regions which contain the latch, threonine at p+2 closes the latch and restricts the motion of the hinge. The ability of serine at p+2 to pin the latch closed is impaired because serine is incapable of forming stable simultaneous interactions with the periplasmic domain and the EL5 loop due to the loss of the methyl group.

**FIGURE 6:**
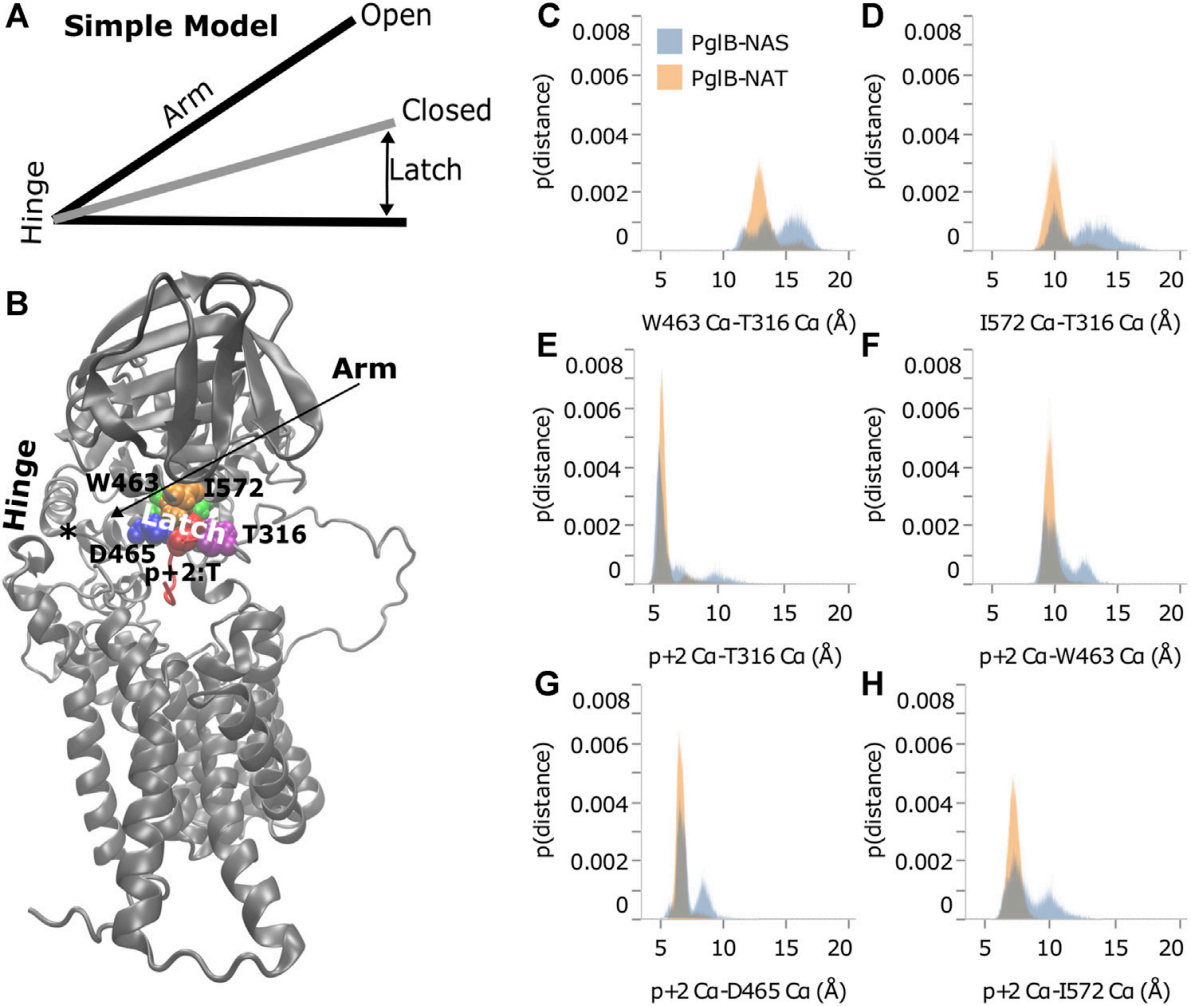
Threonine at p+2 acts as a pin in a latch. **(A)** Simple hinge-latch model. **(B)** Structure of PglB showing hinge (asterisk marks the junction of the two helices at N448 and Y473), arm, latch: W463 (green), D465 (blue), I572 (orange), T316 (purple), and pin residue p+2 (red). Histograms of Cα-Cα distances between PglB latch residues **(C,D)** and between PglB latch residues and p+2 pin residue **(E–H)** for PglB-NAT (light blue) and PglB-NAS (orange). The probability is the fraction of the total bound frames of PglB-NAT or PglB-NAS, respectively.

As shown in Figure 4C, distance from the hinge (N448/Y473) is strongly correlated with backbone displacement of the periplasmic domain, consistent with a hinge-like motion. We propose that the difference between the two peptides when bound to PglB is consistent with a simple hinge-latch model (Figure 6A). In this model, the pin connects the two sides of the latch when closed. An arm connects the hinge and latch and mediates the restriction of the domain motion when the pin closes the latch. In PglB, N448/Y473 is the hinge, residues W463, D465, I572, and T316 form the two sides of the latch, p+2 acts as the pin to hold the latch closed when threonine, and the helix connecting the hinge and latch is the arm (residues 463–472) (Figure 6B).

If the hinge-latch model is accurate for PglB, the motion should be different when latched (PglB-NAT) versus unlatched (PglB-NAS). The motion should be more restrained in PglB-NAT and the motion should be freer in PglB-NAS. Principal component analysis of PglB (excluding the unstructured EL5 N-terminus) shows that the motion of PglB-NAT and PglB-NAS is similar (Supplementary Figures S2, S3). However, the frequency of sampling different regions of the conformational space is different (Supplementary Figure S6). PglB-NAT is more concentrated in the center of the conformational space, which is consistent with restrained motion of the hinge when the latch is pinned (Supplementary Figure S6A). PglB-NAS is more broadly distributed with higher sampling in the tails, which is indicative of increased motion consistent with an unlatched hinge (Supplementary Figure S6B).

The motion of PglB-NAT versus PglB-NAS supports the existence of a latch. To verify that our choice of latch and pin residues is correct, we looked at the distance between these residues in the latched (PglB-NAT) and unlatched (PglB-NAS) conditions. For the latch and pin to restrict the motion of the hinge (as we propose occurs in PglB-NAT), the latch and pin complex must be stable (the distances between these residues should not fluctuate significantly). When the pin is unable to close the latch (as we propose occurs in PglB-NAS), we expect to see greater fluctuations in the distance between the pin and latch residues. The histograms of the distances between the PglB residues on opposite sides of the latch (W463 and I572 of the periplasmic domain with T316 of the EL5 loop) show that the latch residues in PglB-NAT predominantly sample a narrow range of distances (Figures 6C,D, gray). The distances between the pin (p+2) and the latch residues show a similar preference for sampling a restricted range of distances (Figures 6E–H, gray). In PglB-NAS, the distances between the latch and pin residues sample larger distances more often than in PglB-NAT (Figures 6C–H, red). These results show that when p+2 is threonine, the latch/pin complex is stable, as required to restrict the motion of the hinge in the hinge-latch model. The increased sampling of larger distances between the pin and latch residues in PglB-NAS is instead consistent with a pin that is unable to keep the latch closed.

The behavior of the pin and latch residues explains the interplay between peptide binding and periplasmic domain orientation. The distances between the latch residues in PglB-NAS increase because serine lacks the methyl group and is unable to form the stable simultaneous interactions necessary to pin the latch closed (keeping the two sides of the latch in closer proximity). The NAS peptide therefore shifts its position to interact with the latch residues on opposite sides (periplasmic domain and EL5) because the latch residues are further apart. As the NAS peptide moves towards one side of the latch, it loses the interaction with the other side of the latch (reflected in the increased sampling of near-zero interaction energies (Supplementary Figure S9). The increased distance between the latch residues and the subsequent motion of the NAS peptide explain the weakened H-bonds important for substrate binding because these bonds between the enzyme and substrate overlap with the latch/pin complex (Table 1 and Supplementary Table S5). Of the six H-bonds identified as important for a stably bound peptide, four involve the PglB latch residues, two involve neighboring residues, and both sides of the latch are important (four H-bonds with the periplasmic domain and two H-bonds with EL5). Similarly for the substrate, of these six H-bonds, four involve the pin residue (p+2) and two involve adjacent substrate residues (p+1/p+3). For these reasons, the state of the latch (pinned or unpinned) is inextricably linked to the partial unbinding of the substrate.

### Substrate Mediated Allosteric Networks

**FIGURE 7:**
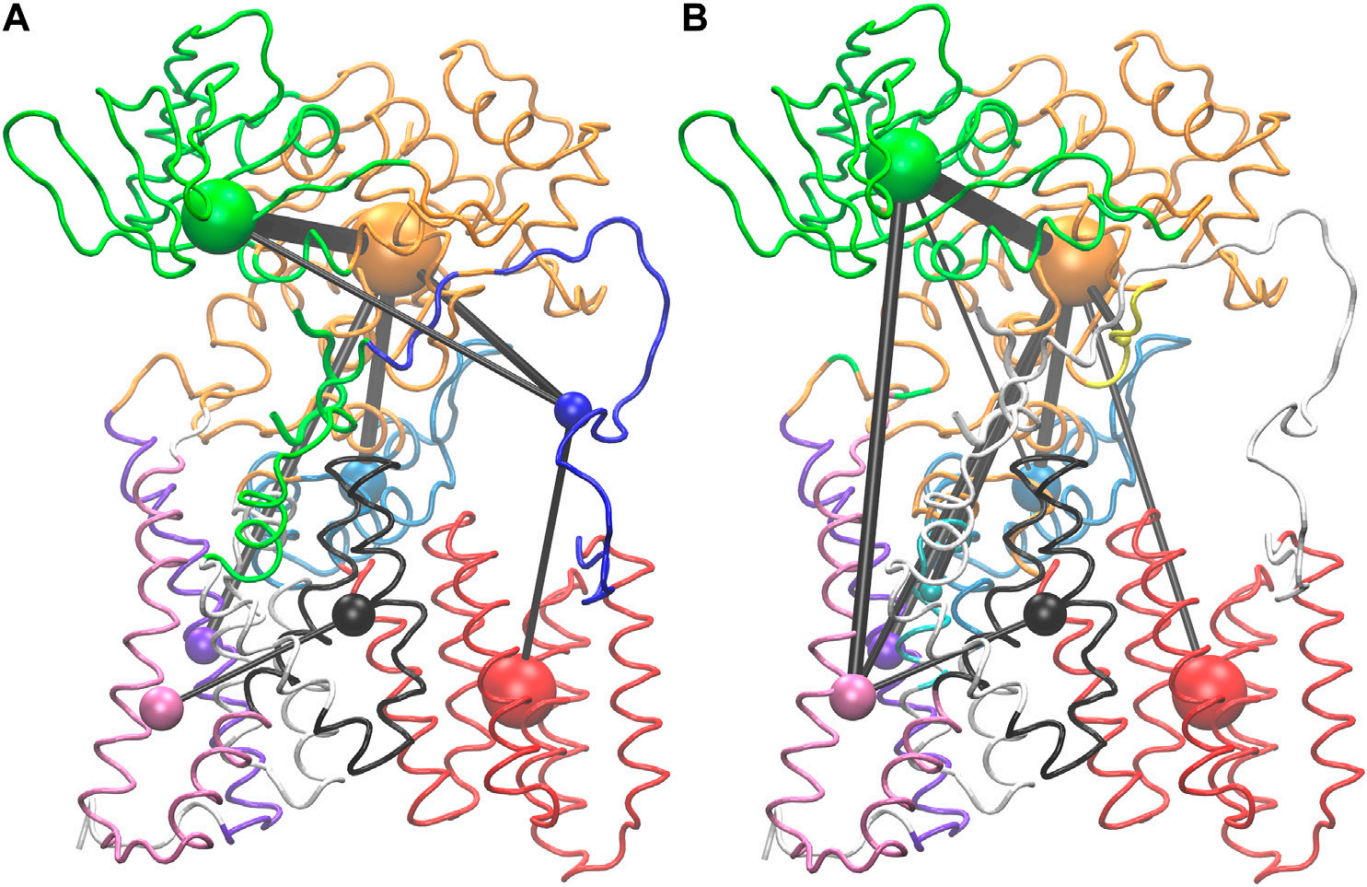
Visualization of PglB networks in PglB-NAT **(A)** and PglB-NAS **(B)**. Residues are colored according to community membership; the corresponding colored sphere is proportional to the community size and the edge thickness is a function of the correlation strength. Residues of unconnected communities are colored white.

Network analysis was used to look for allosteric communication mediated by the peptide as suggested by the hinge and latch model. The network of PglB-NAT consists of eight communities while the PglB-NAS network contains nine communities (Figure 7, Supplementary Figure S7). Seven of these communities are analogous in PglB-NAT and PglB-NAS with 60–100% similarity containing 626 residues of PglB-NAT and 603 residues of PglB-NAS (out of 711 PglB residues, 8 peptide residues, and 2 Mg^2+^ ions). The two largest communities contain most of the periplasmic domain residues (Figure 7, Supplementary Figure S7: orange, green) and are 60–70% analogous. The third largest community (transmembrane helices 4–9; Figure 7, Supplementary Figure S7: red) is 98% analogous in the two networks and is consistent with previous results that showed these helices move as a single unit to accommodate the presence of LLO (Lee and Im, 2017). The last four analogous communities contain 30–50 residues each and are comprised of the EL1 residues (Figure 7, Supplementary Figure S7: light blue) and subsets of the remaining transmembrane helices (Figure 7, Supplementary Figure S7: black, purple, pink). Of the residues not found in the analogous communities, 32 PglB-NAT residues are part of a community not connected in the network of PglB-NAS (Figure 7, Supplementary Figure S7: blue, EL5) and 11 PglB-NAS residues are part of a community not connected in the network of PglB-NAT (Figure 7, Supplementary Figure S7: cyan, transmembrane helix). An additional five residues (483–487) containing part of the bacterial DGGK motif (residues 481–484; Supplementary Figure S1C, cyan), which is suggested to be important for promoting proper conformation of the p0 side chain for LLO attachment (Pedebos et al., 2015; Napiórkowska et al., 2017), is part of a separate community (Figure 7, Supplementary Figure S7: yellow) in PglB-NAS instead of the large community containing the WWD motif as in PglB-NAT (Figure 7, Supplementary Figure S7: orange).

Lastly, 63 PglB-NAT residues and 102 PglB-NAS residues are unconnected (Figure 7, Supplementary Figure S7: white). In PglB-NAT, the residues in unconnected communities are part of the transmembrane domain. These residues include the N-terminal tail and residues around the stable core (see *Analysis of Trajectories*). Half of the unconnected residues in PglB-NAS are similarly located in the transmembrane domain. Significantly, the remaining unconnected residues in PglB-NAS are the entire EL5 loop (43 residues) and the peptide (8 residues) (Figure 7, Supplementary Figure S7: white). In PglB-NAT, the EL5 loop is part of three communities: blue (residues 283–309, 311–315), orange (residue 310) and green (residues 316–324) (Figure 7, Supplementary Figure S7). The NAT peptide is also part of the green community, which contains most of the residues important for both peptide binding and the pin and latch behavior demonstrated for p+2:T (see Figure 5A and Table 4), excluding only the WWD motif which is located in the adjacent, strongly connected orange community in both PglB-NAT and PglB-NAS (Figure 7, Supplementary Figure S7). The lack of edges connecting the communities containing the peptide and EL5 in PglB-NAS suggests that allosteric communication between the periplasmic domain and EL5, mediated by the peptide, is lost in the PglB-NAS complex.

**FIGURE 8:**
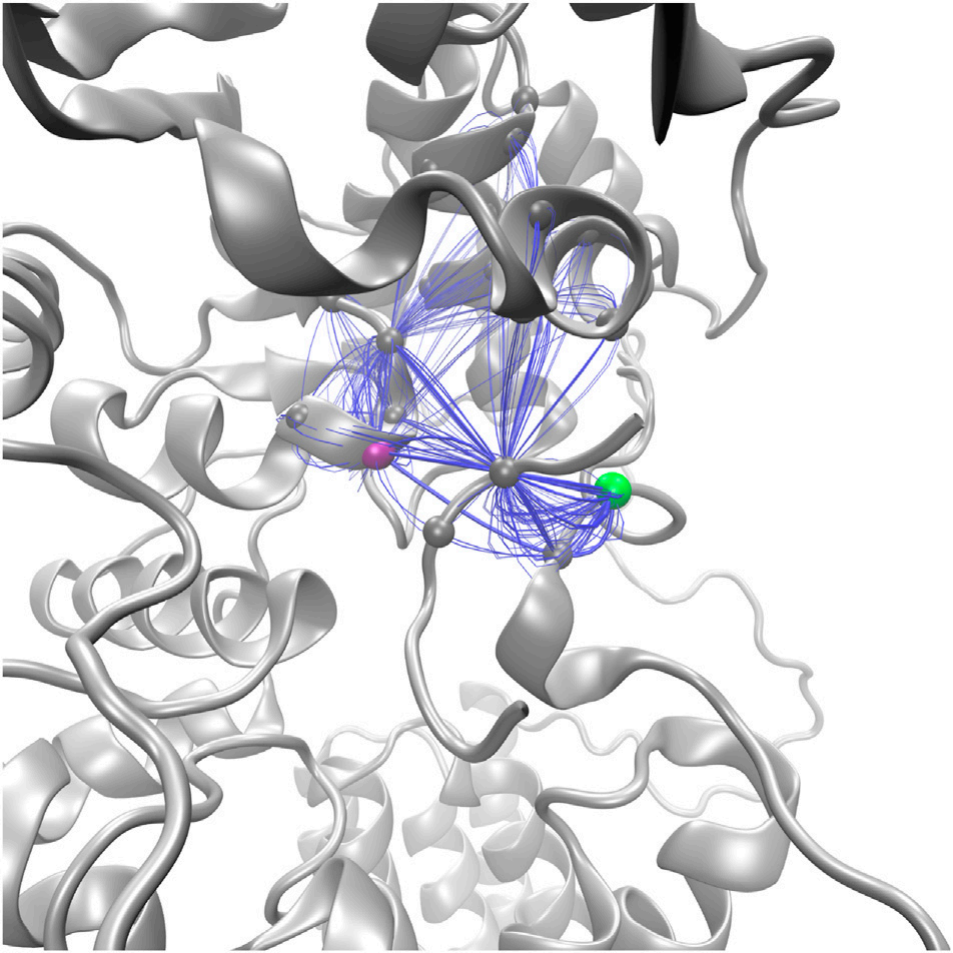
Top 100 suboptimal paths (blue lines) in the network of PglB-NAT connecting D465 (purple, periplasmic) with T316 (green, EL5).

Suboptimal path analysis can show paths of allosteric communication between different parts of the network (Scarabelli and Grant, 2014; Yao et al., 2016). The periplasmic latch residues (W463, D465, and I572) are connected to the EL5 latch residue (T316) by a diverse network of paths passing through the pin (p+2, 99.6% of paths) (Figure 8). Network attributes such as the number of edges per node (degree) and the number of shortest paths that pass through each node (betweenness) further illustrate the high connectivity of the NAT peptide and that adjacent peptide residues p+1 and p+3 play a supporting role in the network of PglB-NAT (Supplementary Table S9). In contrast, at the threshold used for the network analysis (cross-correlation >0.7), no paths connect the periplasmic domain and EL5 in PglB-NAS (Figure 7). Since a threshold imposes an arbitrary cutoff, we examined the cross-correlation values between the three most important peptide residues in the network of PglB-NAT (p+1, p+2, and p+3) and the latch residues (W463, D465, I572, and T316). Only three residue pairs have cross-correlation values near the threshold in PglB-NAS (cross correlation >0.68): p+1 with W463 and W464 and p+2 with the adjacent I317 (Supplementary Table S10). In PglB-NAT, 12 out of 18 pairs meet the 0.7 threshold and 15 out of 18 pairs meet the 0.68 threshold (Supplementary Table S10); the cross-correlation cutoff would have to be lowered to 0.51 in PglB-NAS to match the level of connectivity in the network of PglB-NAT (Supplementary Table S10). These results support that the allosteric communication between the periplasmic domain and EL5 is disrupted in PglB-NAS because the NAS peptide does not mediate the interaction effectively.

### Comparison of the Substrate Binding Pocket of PglB and Human OST

In the cryo-EM structure of the human OST complex with acceptor peptide (OST-B, PDB code 6S7T (Ramírez et al., 2019), the p+2 binding pocket is structurally similar to the binding pocket in PglB (Supplementary Figure S10), thus our findings are likely generalizable to the eukaryotic OSTs. However, a lysine residue (K674) is present in the analogous position of I572 in the structure of PglB (Supplementary Figure S10, orange). The cryo-EM structure suggests that K674 in the human OST may form a hydrogen bond with D606 of the WWD motif (Supplementary Figure S9, blue). This H-bond would stabilize the nearby tryptophan (W677), which is structurally analogous to V575 in the extended bacterial MIV motif (Maita et al., 2010) (Supplementary Figure S10, yellow). The methyl group of p+2 is rotated towards W677 in the human structure (Supplementary Figure S10, red/yellow), suggesting W677 replaces the stabilizing van der Waals interaction formed with I572 in the bacterial structure. W677 is conserved in eukaryotes (Supplementary Figure S1D) as part of an extended hydrophobic motif after the previously identified DXXK motif (Maita et al., 2010). W677 also stacks with S402 (of the SVSE motif, Supplementary Figure S1B) in the structurally analogous position to T316 (Supplementary Figure S10, purple), possibly explaining the conservation of the smaller serine in this position for eukaryotes (Jaffee and Imperiali, 2011). Overall, the structural similarity between the binding pockets suggests that a similar hinge/latch mechanism is present in eukaryotic OSTs with variations that modulate the catalytic efficiency for serine-containing substrates.

## Discussion and Conclusion

The crystal structure of *C. lari* PglB shed light on the catalytic mechanism of asparagine-linked glycosylation and provided a structural explanation for the difference in catalytic efficiency between N-X-T and N-X-S substrates (where X can be any residue but proline in the consensus sequence): the loss of the methyl group would eliminate the van der Waals interaction between the substrate residue at position p+2 and I572 of PglB (Lizak et al., 2011). However, there remained four hydrogen bonds between the p+2 substrate residue and the WWD motif, making the van der Waals interaction with I572 seemingly a relatively small contribution. In this paper, we focused on the substrate bound phase of the glycosylation mechanism of PglB to explore how the two different residues at position p+2 (threonine and serine) could affect the catalytic competency. We found that the two substrates exhibited a striking difference: the substrate peptide containing serine was 2.5 times more likely to experience partial unbinding over 500 ns than the substrate peptide containing threonine. While striking, this is supported by the different binding affinities measured for the NAT and NAS peptides. The peptide binding affinity for PglB is 4 times lower for NAS peptides compared to NAT peptides (Gerber et al., 2013), indicative of a higher off-rate for NAS over NAT peptides.

Examining the PglB-peptide interactions while bound, we identified multiple hydrogen bonds that are important for stable binding–previously identified interactions with the WWD motif and newly identified interactions with the structured EL5 C-terminus. PglB when bound to the NAS peptide sampled a greater range of conformations and exhibited decreased formation of these key hydrogen bonds compared to PglB when bound to the NAT peptide. Despite this, the catalytically relevant H-bonds between the acceptor asparagine and D56/E319 had similar transient formation for the two peptides in the absence of LLO (necessary for a catalytically competent state). However, since being bound in the catalytic pocket is a prerequisite to the formation of these H-bonds, the increased partial substrate disassociation observed for PglB-NAS decreased the population of catalytically competent states by roughly half.

**FIGURE 9:**
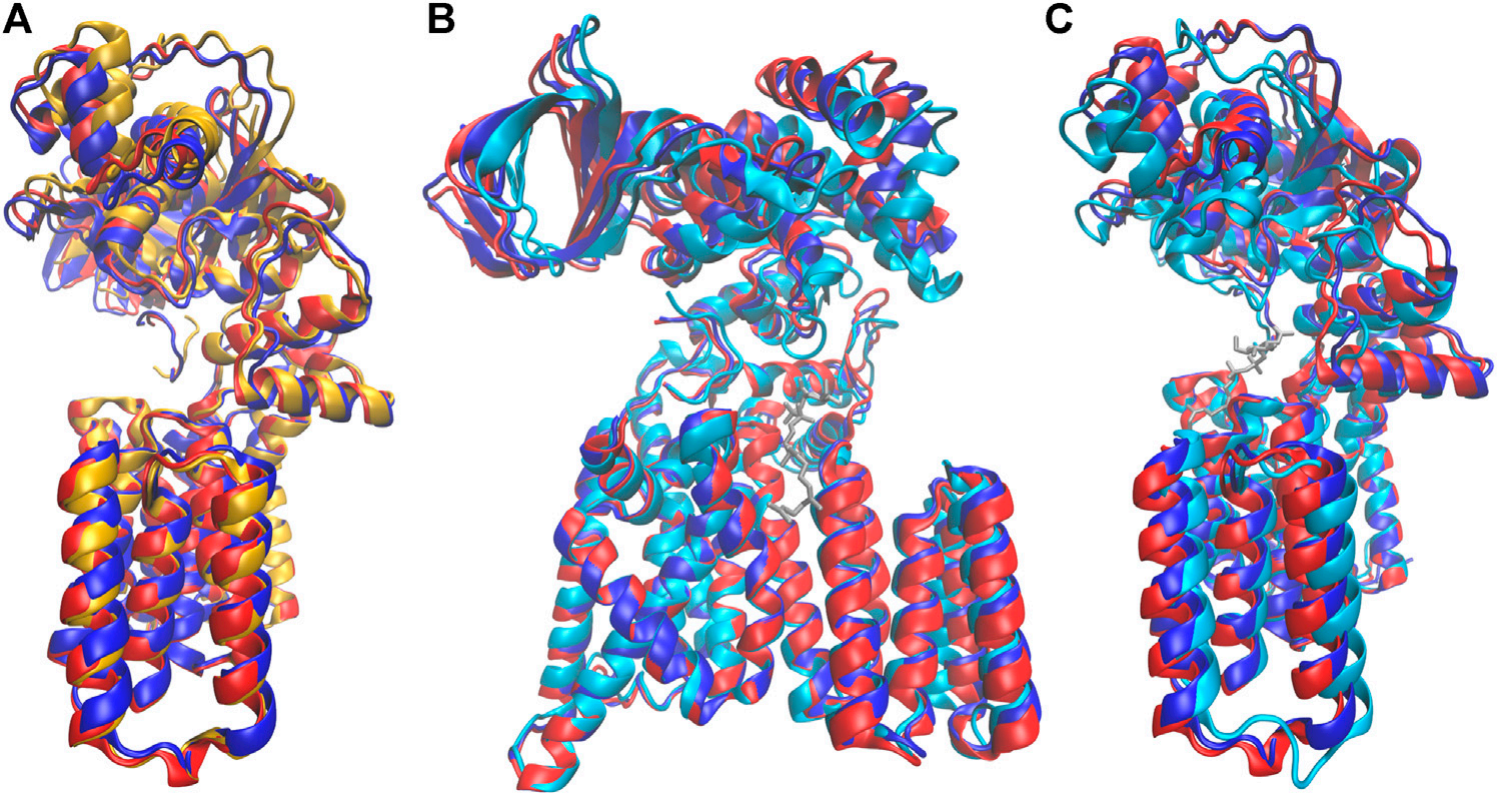
Comparison of structures at different states in the catalytic cycle. Average bound structure of PglB-NAT (blue) and PglB-NAS (red) aligned on consensus stable core residues compared with **(A)** the average unbound structure from PglB-NAS (yellow) and **(B,C)** the crystal structure of PglB in complex with the acceptor peptide and an LLO analog (cyan, LLO analog in gray), front **(B)** and side **(C)** views (PDB code: 5OGL). EL5 not shown for clarity.

More remarkably, the identity of the amino acid at the p+2 position of the substrate peptide also affected the periplasmic domain orientation of PglB. The two bound structures are similar, but they differ in ways that suggest either the unbound or the peptide/LLO bound states. Comparison of the average bound structures of PglB-NAT and PglB-NAS with the average unbound structure (Figure 9A) shows that the average structure of bound PglB-NAS more closely resembles the more open unbound structure than does the average structure of bound PglB-NAT. More open periplasmic domain conformations have been observed in the absence of substrates and were hypothesized to play a role in product release (Pedebos et al., 2015). In contrast, comparison of the average peptide-bound structures with the crystal structure of the PglB-peptide-LLO complex (Napiórkowska et al., 2017) (Figures 9B,C) shows that the periplasmic domain is oriented such that the binding pocket between the periplasmic and transmembrane domains is more compact when the LLO analog is present. This suggests that a more closed conformation of the periplasmic domain is important for a catalytically competent state, and thus the more closed periplasmic domain orientation of PglB-NAT compared to PglB-NAS may play a role in their different catalytic competencies.

Rigid body domain motion was found to describe the difference between the conformations of the periplasmic domain in PglB-NAT and PglB-NAS with a hinge near the junction of the two helices containing N448 and Y473, near the interface of the periplasmic and transmembrane domains. Threonine at position +2 acts as a pin in a latch to inhibit the hinge motion by forming interactions with residues in the periplasmic domain and EL5. Loss of the methyl group in serine results in the disruption of these interactions, allowing the latch to open. The pin/latch complex and the H-bonds necessary for stable substrate binding involve many of the same residues. We found that the opening of the latch necessarily disrupts the binding, and thus combined with the more open periplasmic domain orientation produces the fundamental difference observed between the simulations of PglB-NAT and PglB-NAS, namely, the 2.5 times increase in the frequency of partial NAS peptide unbinding. The importance of the newly identified substrate-EL5 interaction is supported by, and may help explain, the conservation of T316 as part of the bacterial TIXE motif (Jaffee and Imperiali, 2011). Network analysis confirmed the existence of allosteric communication mediated by the peptide between the periplasmic domain and EL5 for NAT peptides and showed that it is greatly weakened for NAS peptides.

Neighboring substrate residues at positions p+1 and p+3 play supporting roles in the binding and allosteric communication. Both p+1 and p+3 form strong H-bonds in PglB-NAT (*p* > 0.8) that we identified as important for substrate binding stability. Substrate residues p+1 and p+3 form backbone H-bonds with the structured portion of EL5; EL5 pins the substrate in the binding pocket and p+1/p+3 play a role in stabilizing the peptide-EL5 interaction. We also found a robust network of interactions between the periplasmic domain and EL5 mediated by the NAT peptide with high connectivity of multiple substrate residues (including p+1 and p+3), facilitating allosteric communication.

These results offer potential explanations for the observation that the catalytic competency of N-X-S substrates is more sensitive to nearby residues p+1 and p+3 than N-X-T substrates (Mellquist et al., 1998; Malaby and Kobertz, 2014). With the disruption of the H-bonds with the WWD motif, the p+1 and p+3 H-bonds with EL5 may play a larger role in keeping serine-containing substrates bound in the catalytic pocket. Intriguingly, the degree to which the p+1 and p+3 interactions are affected in our simulations mirrors their observed effect on the catalytic competency. In our substrate peptides, p+1 is an alanine [found in better substrates (Malaby and Kobertz, 2014)] and p+3 is a phenylalanine [found in worse substrates (Mellquist et al., 1998)]. Despite having more than three times the number of edges (p+1 has 15 vs. 52 edges for p+3), the number of shortest paths that pass through p+3 is a fraction of the shortest paths that pass through p+1 (46 vs. 2,268 for p+1 and p+3, respectively), indicating that p+1 is far more robustly connected in the network of PglB-NAT. While the p+1 and p+3 H-bonds with EL5 form with similar probability in PglB-NAT, this difference in substrate residue connectivity appears to have a more functional affect in PglB-NAS, where the p+1-M318 H-bond probability is decreased by 0.22 but the p+3-T316 H-bond probability is decreased by 0.45, more than twice the decrease of the p+1 H-bond. Similarly, in PglB-NAS the cross-correlations between the peptide and key PglB residues decrease on average compared to PglB-NAT by half as much for p+1 compared to p+2 and p+3 (a decrease of 0.1 for p+1 vs. 0.2 for p+2/p+3).

Experimental data have shown that an interplay exists between the catalytic competency and the substrate residues at positions +1, +2, and +3–serine at p+2 increases the sensitivity to p+1 and p+3, and a less optimal residue at p+1 was found to make differences at p+3 more pronounced (Mellquist et al., 1998; Malaby and Kobertz, 2014). This observation is consistent with our simulation results that show a dynamic network between substrate and enzyme: the strong network of interactions with p+2 when threonine is present leads to more stable substrate-enzyme binding, facilitating the formation of interactions between PglB and p+1/p+3, even with less optimal residues; with serine, this dominant interaction is weakened and the supporting roles of p+1/p+3 become more important to the catalytic competency and thus deficiencies are more consequential.

Certain features that play a role in the behavior observed in our simulations are known to differ between bacterial and eukaryotic OSTs and may contribute to greater sensitivity to serine-containing substrates in eukaryotes (Bause, 1984; Breuer et al., 2001; Chen et al., 2007; Gerber et al., 2013). We found that the conserved D at the p-2 position in the longer bacterial consensus sequence [D-X_-1_-N-X_+1_-S/T, where X_-1_ and X_+1_ can be any residue except proline (Young et al., 2006)] compensates partially for the lost favorable interactions in serine substrates; without the compensation offered by the aspartic acid at p-2 the difference in the interaction energy with PglB between NAT and NAS peptides would be almost double. In eukaryotic OSTs, the interaction at position p-2 with the asparagine residues of the transmembrane domain is not conserved, which may explain why eukaryotic OSTs have greatly reduced catalytic activity for N-X-S peptides (Bause, 1984; Breuer et al., 2001) while bacterial OSTs have a more modest difference in comparison (Chen et al., 2007; Gerber et al., 2013).

Van der Waals interactions between p+2 and T316 are part of the network of interactions that facilitate allosteric communication between the periplasmic domain and EL5. Although this interaction is directly affected by the lost methyl group, the NAS peptide was able to still form transient interactions with T316. T316 is part of the conserved TIXE motif containing the catalytic E319 in bacteria; in eukaryotes, a different sequence is conserved: SVSE (Jaffee and Imperiali, 2011) (Supplementary Figure S1B). The replacement of threonine by the smaller serine in this sequence would further compromise the ability of serine-containing substrates to form stable interactions with EL5 in eukaryotes.

From our results we conclude that the loss of the methyl group in serine has repercussions far beyond the van der Waals interaction with I572 observed in threonine substrates. The loss of the methyl group destabilizes the entire constellation of interactions present with threonine and disrupts neighboring interactions between substrate and enzyme. This interrupts the allosteric communication between the periplasmic domain and EL5 mediated by acceptor substrates with threonine, allowing for opening of the periplasmic domain. While threonine at the +2 position acts as a pin in a latch to favor more closed periplasmic domain orientations reminiscent of the crystal structure of PglB with acceptor substrate and LLO analog (Napiórkowska et al., 2017), the more open periplasmic domain orientations seen with serine likely combine with the weakened substrate-enzyme interactions to increase substrate release and thereby decrease the formation of catalytically competent states.

## Data Availability

The raw data supporting the conclusion of this article will be made available by the authors, without undue reservation.

## References

[B1] Aebi M. (2013). N-linked Protein Glycosylation in the ER. Biochim. Biophys. Acta Mol. Cel Res. 1833, 2430–2437. 10.1016/j.bbamcr.2013.04.001 23583305

[B2] Bai L. Wang T. Zhao G. Kovach A. Li H. (2018). The Atomic Structure of a Eukaryotic Oligosaccharyltransferase Complex. Nature 555, 328–333. 10.1038/nature25755 29466327 PMC6112861

[B3] Barre Y. Nothaft H. Thomas C. Liu X. Li J. Ng K. K. (2017). A Conserved DGGK Motif Is Essential for the Function of the PglB Oligosaccharyltransferase from Campylobacter Jejuni. Glycobiology 27, 978–989. 10.1093/glycob/cwx067 28922740

[B4] Bause E. (1984). Model Studies on N-Glycosylation of Proteins. Biochem. Soc. Trans. 12, 514–517. 10.1042/bst0120514 6428943

[B5] Blum M. Chang H.-Y. Chuguransky S. Grego T. Kandasaamy S. Mitchell A. (2021). The InterPro Protein Families and Domains Database: 20 Years on. Nucleic Acids Res. 49, D344–D354. 10.1093/nar/gkaa977 33156333 PMC7778928

[B6] Braunger K. Pfeffer S. Shrimal S. Gilmore R. Berninghausen O. Mandon E. C. (2018). Structural Basis for Coupling Protein Transport and N-Glycosylation at the Mammalian Endoplasmic Reticulum. Science 360, 215–219. 10.1126/science.aar7899 29519914 PMC6319373

[B7] Breuer W. Klein R. A. Hardt B. Bartoschek A. Bause E. (2001). Oligosaccharyltransferase Is Highly Specific for the Hydroxy Amino Acid in Asn-Xaa-Thr/Ser. FEBS Lett. 501, 106–110. 10.1016/S0014-5793(01)02641-2 11470266

[B8] Chen M. M. Glover K. J. Imperiali B. (2007). From Peptide to Protein: Comparative Analysis of the Substrate Specificity of N-Linked Glycosylation in C. Jejuni. Biochemistry 46, 5579–5585. 10.1021/bi602633n 17439157

[B9] Clauset A. Newman M. E. J. Moore C. (2004). Finding Community Structure in Very Large Networks. Phys. Rev. E 70, 6–2. 10.1103/PhysRevE.70.066111 15697438

[B10] Eichler J. (2013). Extreme Sweetness: Protein Glycosylation in Archaea. Nat. Rev. Microbiol. 11, 151–156. 10.1038/nrmicro2957 23353769

[B11] Essmann U. Perera L. Berkowitz M. L. Darden T. Lee H. Pedersen L. G. (1995). A Smooth Particle Mesh Ewald Method. J. Chem. Phys. 103, 8577–8593. 10.1063/1.470117

[B12] Fiser A. Do R. K. G. Šali A. (2000). Modeling of Loops in Protein Structures. Protein Sci. 9, 1753–1773. 10.1110/ps.9.9.1753 11045621 PMC2144714

[B13] Fraley C. Raftery A. E. (2002). Model-based Clustering, Discriminant Analysis and Density Estimation. J. Am. Stat. Assoc. 97, 611. 10.1198/016214502760047131

[B14] Frishman D. Argos P. (1995). Knowledge-based Protein Secondary Structure Assignment. Proteins 23, 566–579. 10.1002/prot.340230412 8749853

[B15] Gerber S. Lizak C. Michaud G. Bucher M. Darbre T. Aebi M. (2013). Mechanism of Bacterial Oligosaccharyltransferase. J. Biol. Chem. 288, 8849–8861. 10.1074/jbc.M112.445940 23382388 PMC3610960

[B16] Grant B. J. Rodrigues A. P. C. ElSawy K. M. McCammon J. A. Caves L. S. D. (2006). Bio3d: an R Package for the Comparative Analysis of Protein Structures. Bioinformatics 22, 2695–2696. 10.1093/bioinformatics/btl461 16940322

[B17] Helenius A. Aebi M. (2004). Roles of N-Linked Glycans in the Endoplasmic Reticulum. Annu. Rev. Biochem. 73, 1019–1049. 10.1146/annurev.biochem.73.011303.073752 15189166

[B18] Humphrey W. Dalke A. Schulten K. (1996). VMD: Visual Molecular Dynamics. J. Mol. Graph. 14, 33–38. 10.1016/0263-7855(96)00018-5 8744570

[B19] Igura M. Maita N. Kamishikiryo J. Yamada M. Obita T. Maenaka K. (2008). Structure-guided Identification of a New Catalytic Motif of Oligosaccharyltransferase. EMBO J. 27, 234–243. 10.1038/sj.emboj.7601940 18046457 PMC2206122

[B20] Ihssen J. Kowarik M. Wiesli L. Reiss R. Wacker M. Thöny-Meyer L. (2012). Structural Insights from Random Mutagenesis of Campylobacter Jejuni Oligosaccharyltransferase PglB. BMC Biotechnol. 12, 67. 10.1186/1472-6750-12-67 23006740 PMC3527161

[B21] Jaffee M. B. Imperiali B. (2011). Exploiting Topological Constraints to Reveal Buried Sequence Motifs in the Membrane-Bound N-Linked Oligosaccharyl Transferases. Biochemistry 50, 7557–7567. 10.1021/bi201018d 21812456 PMC3164389

[B22] Jorgensen W. L. Chandrasekhar J. Madura J. D. (1983). Comparison of Simple Potential Functions for Simulating Liquid Water. J. Chem. Phys. 79, 926. 10.1063/1.445869

[B23] Kelleher D. J. Gilmore R. (2006). An Evolving View of the Eukaryotic Oligosaccharyltransferase. Glycobiology 16, 47R–62R. 10.1093/glycob/cwj066 16317064

[B24] Kowarik M. Young N. M. Numao S. Schulz B. L. Hug I. Callewaert N. (2006). Definition of the Bacterial N-Glycosylation Site Consensus Sequence. EMBO J. 25, 1957–1966. 10.1038/sj.emboj.7601087 16619027 PMC1456941

[B25] Laskowski R. A. MacArthur M. W. Moss D. S. Thornton J. M. (1993). PROCHECK: a Program to Check the Stereochemical Quality of Protein Structures. J. Appl. Cryst. 26, 283–291. 10.1107/S0021889892009944

[B26] Lee H. S. Im W. (2017). Transmembrane Motions of PglB Induced by LLO Are Coupled with EL5 Loop Conformational Changes Necessary for OST Activity. Glycobiology 27, 787. 10.1093/glycob/cwx059 31972022

[B27] Li L. Woodward R. Ding Y. Liu X.-w. Yi W. Bhatt V. S. (2010). Overexpression and Topology of Bacterial Oligosaccharyltransferase PglB. Biochem. Biophys. Res. Commun. 394, 1069–1074. 10.1016/j.bbrc.2010.03.126 20331969

[B28] Lizak C. Gerber S. Numao S. Aebi M. Locher K. P. (2011). X-ray Structure of a Bacterial Oligosaccharyltransferase. Nature 474, 350–355. 10.1038/nature10151 21677752

[B29] Lizak C. Gerber S. Zinne D. Michaud G. Schubert M. Chen F. (2014). A Catalytically Essential Motif in External Loop 5 of the Bacterial Oligosaccharyltransferase PglB. J. Biol. Chem. 289, 735–746. 10.1074/jbc.M113.524751 24275651 PMC3887201

[B30] Lohr S. L. (1999). Sampling: Design and Analysis. California: Duxbury Press.

[B31] MacKerell A. D. Bashford D. Bellott M. Dunbrack R. L. Evanseck J. D. Field M. J. (1998). All-Atom Empirical Potential for Molecular Modeling and Dynamics Studies of Proteins†. J. Phys. Chem. B 102 (18), 3586–3616. 10.1021/jp973084f 24889800

[B32] Madeira F. Park Y. M. Lee J. Buso N. Gur T. Madhusoodanan N. (2019). The EMBL-EBI Search and Sequence Analysis Tools APIs in 2019. Nucleic Acids Res. 47, W636–W641. 10.1093/nar/gkz268 30976793 PMC6602479

[B33] Maita N. Nyirenda J. Igura M. Kamishikiryo J. Kohda D. (2010). Comparative Structural Biology of Eubacterial and Archaeal Oligosaccharyltransferases. J. Biol. Chem. 285, 4941–4950. 10.1074/jbc.M109.081752 20007322 PMC2836098

[B34] Malaby H. L. H. Kobertz W. R. (2014). The Middle X Residue Influences Cotranslational N-Glycosylation Consensus Site Skipping. Biochemistry 53, 4884–4893. 10.1021/bi500681p 25029371 PMC4372077

[B35] Mellquist J. L. Kasturi L. Spitalnik S. L. Shakin-Eshleman S. H. (1998). The Amino Acid Following an Asn-X-Ser/Thr Sequon Is an Important Determinant of N-Linked Core Glycosylation Efficiency. Biochemistry 37, 6833–6837. 10.1021/bi972217k 9578569

[B36] Mohorko E. Glockshuber R. Aebi M. (2011). Oligosaccharyltransferase: the central Enzyme of N-Linked Protein Glycosylation. J. Inherit. Metab. Dis. 34, 869–878. 10.1007/s10545-011-9337-1 21614585

[B37] Napiórkowska M. Boilevin J. Sovdat T. Darbre T. Reymond J.-L. Aebi M. (2017). Molecular Basis of Lipid-Linked Oligosaccharide Recognition and Processing by Bacterial Oligosaccharyltransferase. Nat. Struct. Mol. Biol. 24, 1100–1106. 10.1038/nsmb.3491 29058712

[B38] Nothaft H. Szymanski C. M. (2013). Bacterial Protein N-Glycosylation: New Perspectives and Applications. J. Biol. Chem. 288, 6912–6920. 10.1074/jbc.R112.417857 23329827 PMC3591601

[B39] Pedebos C. Arantes P. R. Giesel G. M. Verli H. (2015). In silicoInvestigation of the PglB Active Site Reveals Transient Catalytic States and Octahedral Metal Ion Coordination. Glycobiology 25, 1183–1195. 10.1093/glycob/cwv053 26220543

[B40] Phillips J. C. Braun R. Wang W. Gumbart J. Tajkhorshid E. Villa E. (2005). Scalable Molecular Dynamics with NAMD. J. Comput. Chem. 26 (16), 1781–1802. 10.1002/jcc.20289 16222654 PMC2486339

[B41] Ramírez A. S. Kowal J. Locher K. P. (2019). Cryo-electron Microscopy Structures of Human Oligosaccharyltransferase Complexes OST-A and OST-B. Science 366, 1372–1375. 10.1126/science.aaz3505 31831667

[B42] Romanowska J. Nowiński K. S. Trylska J. (2012). Determining Geometrically Stable Domains in Molecular Conformation Sets. J. Chem. Theor. Comput. 8, 2588–2599. 10.1021/ct300206j 26592104

[B43] Ryckaert J. P. Ciccotti G. Berendsen H. J. C. (1997). Numerical Integration of the Cartesian Equations of Motion of a System with Constraints: Molecular Dynamics of N-Alkanes. J. Comput. Phys. 23, 327–341.

[B44] Scarabelli G. Grant B. J. (2014). Kinesin-5 Allosteric Inhibitors Uncouple the Dynamics of Nucleotide, Microtubule, and Neck-Linker Binding Sites. Biophys. J. 107, 2204–2213. 10.1016/j.bpj.2014.09.019 25418105 PMC4223232

[B45] Szymanski C. M. Burr D. H. Guerry P. (2002). Campylobacter Protein Glycosylation Affects Host Cell Interactions. Infect. Immun. 70, 2242–2244. 10.1128/IAI.70.4.2242-2244.2002 11895996 PMC127875

[B46] Szymanski C. M. Wren B. W. (2005). Protein Glycosylation in Bacterial Mucosal Pathogens. Nat. Rev. Microbiol. 3, 225–237. 10.1038/nrmicro1100 15738950

[B47] Wacker M. Linton D. Hitchen P. G. Nita-Lazar M. Haslam S. M. North S. J. (2002). N-Linked Glycosylation in Campylobacter Jejuni and its Functional Transfer into *E. coli* . Science 298, 1790–1793. 10.1126/science.298.5599.1790 12459590

[B48] Yao X.-Q. Malik R. U. Griggs N. W. Skjærven L. Traynor J. R. Sivaramakrishnan S. (2016). Dynamic Coupling and Allosteric Networks in the α Subunit of Heterotrimeric G Proteins. J. Biol. Chem. 291, 4742–4753. 10.1074/jbc.M115.702605 26703464 PMC4813496

