# Supplementary material for: The Role of Substrate Mediated Allostery in the Catalytic Competency of the Bacterial Oligosaccharyltransferase PglB
Source: Front Mol Biosci. 2021 Sep 15;8:740904. doi: 10.3389/fmolb.2021.740904 (PMC8479172; doi:10.3389/fmolb.2021.740904)
Supplement: Supplementary file 1 [file DataSheet1.pdf]

Table S1. Mean, standard deviation, and standard deviation of the jack-knife means (JK SD) of the peptide-OST interaction energy (kcal/mol)

	Mean	SD	JK SD
PglB-NAT	-231.4	25.3	1.6
PglB-NAS	-224.6	25.3	2.1

Table S2. Mean and standard deviation of distances in the bound state between donor-acceptor of the non-catalytic H-bonds and the proxy for the van der Waals interaction between p+2 and I572.

	PglB-NAT Distance (Å)	PglB-NAS Distance (Å)
p+1 O-M318 N	3.3 ± 0.9	3.9 ± 1.5
p+2 N-D465 Oδ[a.]	3.0 ± 0.6	3.6 ± 1.1
p+2 Oγ-W463 Nε	3.3 ± 0.6	4.6 ± 1.9
p+2 Oγ-W464 Nε	3.8 ± 1.1	5.1 ± 2.0
p+2 Oγ-D465 Oδ[a.]	2.9 ± 0.7	4.1 ± 1.8
p+3 N-T316 O	3.4 ± 1.5	5.2 ± 2.5
p+2 Cβ-I572 Cδ	4.4 ± 0.6	5.7 ± 1.9

[a.] Minimum distance of Oδ1 and Oδ2.

Table S3. Comparison of p+2 interaction energy for subset of PglB residues within 15 Å of p+2 versus all PglB residues

	Subset (kcal/mol)	All PglB (kcal/mol)
Difference, all frames (NAT-NAS)	-14.0	-13.9
Difference, bound (NAT-NAS)	-11.2	-11.1
Total, bound, NAT	-38.3 ± 2.1	-38.3 ± 7.0
Total, bound, NAS	-27.2 ± 1.9	-27.2 ± 11.7

Table S4. Interaction energy between PglB or catalytic Mg^{2+} ion for NAT and NAS peptides by residue

Peptide Residue	PglB-NAT Int. Energy (kcal/mol)	PglB-NAS Int. Energy (kcal/mol)	Mg^{2+}-NAT Int. Energy (kcal/mol)	Mg^{2+}-NAS Int. Energy (kcal/mol)
p-3	-38.1 ± 20.7	-35.1 ± 19.2	36.2 ± 6.7	37.6 ± 6.1
p-2	-54.2 ± 43.7	-64.7 ± 32.4	-51.2 ± 12.2	-53.7 ± 10.3
p-1	-33.2 ± 10.2	-35.2 ± 10.8	6.4 ± 4.7	7.4 ± 5.2
p0	-34.3 ± 17.9	-36.6 ± 13.3	12.2 ± 9.9	12.4 ± 8.8
p+1	-20.1 ± 3.5	-17.1 ± 3.9	-2.1 ± 0.9	-2.03± 1.0
p+2	-38.3 ± 7.0	-27.2 ± 11.7	-0.3 ± 0.5	-0.4 ± 0.9
p+3	-10.3 ± 3.6	-7.6 ± 4.2	0.6 ± 0.5	-0.2 ± 1.1
p+4	-3.0 ± 17.8	-1.2 ± 21.1	-21.0 ± 3.2	-22.7 ± 4.8
total	-231.4 ± 25.3	-224.6 ± 25.3	-19.2 ± 24.6	-21.9 ± 25.6

Table S5. Probability of maintaining or losing the set of three or six H-bonds important for peptide binding for PglB-NAT and PglB-NAS. Starred probabilities include only those trajectories that populate the bound state after the 50 ns equilibration period and initiate unbinding over the 500 ns data collection period (NAT: excludes 9 trajectories, NAS: excludes 3 trajectories). Three non-catalytic H-bonds important for peptide binding: p+1 O-M318 N, p+2 N-D465 Oδ, and p+2 Oγ-W463 Nε. Six non-catalytic H-bonds important for peptide binding: previous three H-bonds plus p+2 Oγ-W464 Nε, p+2 Oγ-D465 Oδ, and p+3 N-T316 O.

Criteria	p(NAT)	p(NAS)	p(NAT*)	p(NAS*)
All 3 non-catalytic H-bonds present	0.68	0.24	0.44	0.13
All 6 non-catalytic H-bonds present	0.24	0.05	0.12	0.02
Loss of all 3 non-catalytic H-bonds	0.007	0.047	0.019	0.081
Loss of all 6 non-catalytic H-bonds	0.003	0.034	0.008	0.059

Table S6. Catalytic H-bond formation in PglB-NAT vs. PglB-NAS. Ratio = N(frames, NAS)/N(frames, NAT) over 10 trajectories each, 450 ns maximum length

Criteria	Ratio (NAS/NAT)
Bound state (includes 50 ns equilibration period)	0.58
Simultaneous catalytically required H-bond formation	0.53
Formation of either catalytically required H-bond	0.56

Table S7. Correlation coefficient (R^2) between distance from residue and backbone RMSD between PglB-NAS and PglB-NAT for residues with low backbone RMSD in the hinge region for the average structures and the most populated structures. Residues 521-550 exhibit increased RMSD relative to their distance from the proposed hinge because the two small helices are connected by long loops to the main periplasmic domain. The correlation coefficients exclude these residues which are more mobile as well as loops/turns in the periplasmic domain.

	Average Structures		Most Populated Structures	
Residue	R^2	Δ BB RMSD (Å)	R^2	Δ BB RMSD (Å)
I52	0.85	0.12	0.81	1.46
T437	0.64	0.10	0.70	1.46
**N448**	0.95	0.19	0.83	2.82
K453	0.87	0.18	0.72	5.81
Y468	0.81	0.45	0.78	0.56
**Y473**	0.95	0.25	0.86	1.99
V699	0.72	0.84	0.59	4.04

Table S8. Correlation coefficient (R^2) between distance from residue and backbone RMSD for periplasmic domain residues (excluding loops/turns and residues 521-550) for residues distant from the proposed hinge (N448/Y473) with low backbone RMSD between PglB-NAS and PglB-NAT (Δ BB RMSD) across the sequence of PglB (average structures and most populated structures)

	Average Structures			Most Populated Structures	
Residue	R^2	Δ BB RMSD (Å)	Distance from N448 (Å)	R^2	Δ BB RMSD (Å)
N15	0.56	0.08	47.5	0.41	0.70
T128	0.39	0.02	47.0	0.26	0.53
M206	0.32	0.58	41.2	0.27	1.57
G345	0.04	0.03	48.9	0.01	0.48
D554	0.26	0.52	31.4	0.15	11.19

Table S9. Number of edges per peptide residue (degree) and number of shortest paths that pass through each peptide residue (betweenness) from networks of PglB-NAT and PglB-NAS

	N_{edge}		N_{paths}	
Peptide Residue	PglB-NAT	PglB-NAS	PglB-NAT	PglB-NAS
p-3	1	1	0	0
p-2	4	4	570	6
p-1	3	3	0	10
p0	3	3	1,704	0
p+1	15	4	2,268	12
p+2	79	3	12,469	10
p+3	52	2	46	6
p+4	43	2	0	0
total	200	22	17,057	44

Table S10. Cross-correlations between pin/latch and adjacent residues for PglB-NAT and PglB-NAS. Bold numbers exceed the threshold used in the network analysis (0.7).

	p+1		p+2		p+3	
PglB Residue	PglB-NAT	PglB-NAS	PglB-NAT	PglB-NAS	PglB-NAT	PglB-NAS
T316	0.695	0.457	**0.772**	0.513	0.664	0.351
I317	**0.801**	0.670	**0.809**	0.689	**0.700**	0.554
W463	**0.701**	0.683	**0.817**	0.644	0.683	0.527
W464	**0.714**	0.692	**0.805**	0.644	0.647	0.473
W465	**0.756**	0.668	**0.824**	0.618	0.667	0.446
I572	0.699	0.662	**0.863**	0.619	**0.821**	0.632
Average	0.728	0.639	0.815	0.621	0.697	0.497

```
A. D56
(B) C. lari              41 FYEFFFND---QLMITTNDGYAFAE--------GA-    64
(B) C. jejuni            39 FNEYFFNN---QLMIISNDGYAFAE--------GA-    62
(A) P. furiosus          46 ---------TAGKYFSDPDTFYHFEIYKLVLKEGL-    71
(A) M. voltae            67 YL-DVFSDDNGRMYLTALDPYYYLRMSENYLENGHT   101
(E) H. sapiens           94 ---------RFESIIHEFDPWFNYRSTHHLASHGFY   120
(E) M. musculus          91 ---------RFESIIHEFDPWFNYRSTHHLASHGFY   117
(E) C. elegans           42 ---------RFESIIHEFDPWFNYRATHHMVQHGFY    68
(E) S. cerevisiae        38 ---------KFESIIHEFDPWFNYRATKYLVNNSFY    64


B. E319
(P) C. lari             306 AAFMYFNVNETIMEVNTI-DPE---VFMQRISSSVL   337
(P) C. jejuni           303 QGFMYFNVNQTIQEVENV-DFS---EFMRRISGSEI   334
(A) P. furiosus         335 GAYQSTQVYETVQELAKT-DWGDVKVYYGVEKPNGI   369
(A) M. voltae           351 SQTGWPNVLTTVSELDTA-SLDEI---ISSSLGSIH   382
(E) H. sapiens          391 YAKIHIPIIASVSEHQPT-TWVS-------------   413
(E) M. musculus         389 YAKIHIPIIASVSEHQPT-TWVS-------------   410
(E) C. elegans          340 YAKIHIPIIASVSEHQPT-TWVS-------------   361
(E) S. cerevisiae       337 YAKIHIPIIASVSEHQPV-SWPA-------------   358


C. WWD Motif, DGGK Motif
(P) C. lari             457 EDYVVAWWDYGYPIRYY--SDVKTLIDGGKHLGKD-   489
(P) C. jejuni           451 EDYVVTWWDYGYPVRYY--SDVKTLVDGGKHLGKD-   483
(A) P. furiosus         505 YSTATSWWDYGYWIESSLLGQRRASADGGHARDRD-   539
(A) M. voltae           586 NSVVTCWWDNGHIYTWK--TDRMVTFDGSSQNTPR-   618
(E) H. sapiens          598 HARVMSWWDYGYQIAGM--ANRTTLVDNNTWNNSHI   631
(E) M. musculus         595 HARVMSWWDYGYQIAGM--ANRTTLVDNNTWNNSHI   628
(E) C. elegans          541 DARVMSWWDYGYQIAGM--ANRTTLVDNNTWNNSHI   574
(E) S. cerevisiae       510 DSKVAAWWDYGYQIGGM--ADRTTLVDNNTWNNTHI   543


D. DXNK/W Motif
(E) H. sapiens          656 YVLVIFGGVIGYSGDDINKFLWMVRIAEGEHPK-DI   690
(E) P. abelii           577 YVLVIFGGLTGYSSDDINKFLWMVRIGGSTDTGKHI   612
(E) B. taurus           577 YVLVIFGGLTGYSSDDINKFLWMVRIGGSTDTGKHI   612
(E) C. lupus            656 YVLVIFGGVIGYSGDDINKFLWMVRIAEGEHPK-DI   690
(E) M. musculus         653 YVLVIFGGVIGYSGDDINKFLWMVRIAEGEHPK-DI   687
(E) C. elegans          599 YILVIFGGVIGYSGDDINKFLWMVRIAQGEHPK-DI   633
(E) S. cerevisiae       568 YVLVIFGGLIGFGGDDINKFLWMIRISEGIWPE-EI   602
(E) S. pombe            602 YILIIYGGTLGYSSDDMNKFLWMIRISQGLWPD-EI   636
(E) D. discoideum       593 YVLVIFGGLTGYSSDDINKFLWMVRIGGSCDPN--I   626
(E) O. sativa           600 YVLVVFGGVTGYSSDDINKFLWMVRIGGGVFPV--I   633
(E) A. thaliana         614 YVLVVFGGVTGYSSDDINKFLWMVRIGGGVFPV--I   647
```

Fig. S1 Sequence alignments with beginning and ending residue numbers showing conservation across families (P, prokaryotes; A, archaea; E, eukaryotes) of catalytically required residues D56 (**A**) and E319 (**B**), the strictly conserved WWD motif and the bacterial DGGK motif (**C**), and the longer hydrophobic motif present in eukaryotes (**D**).

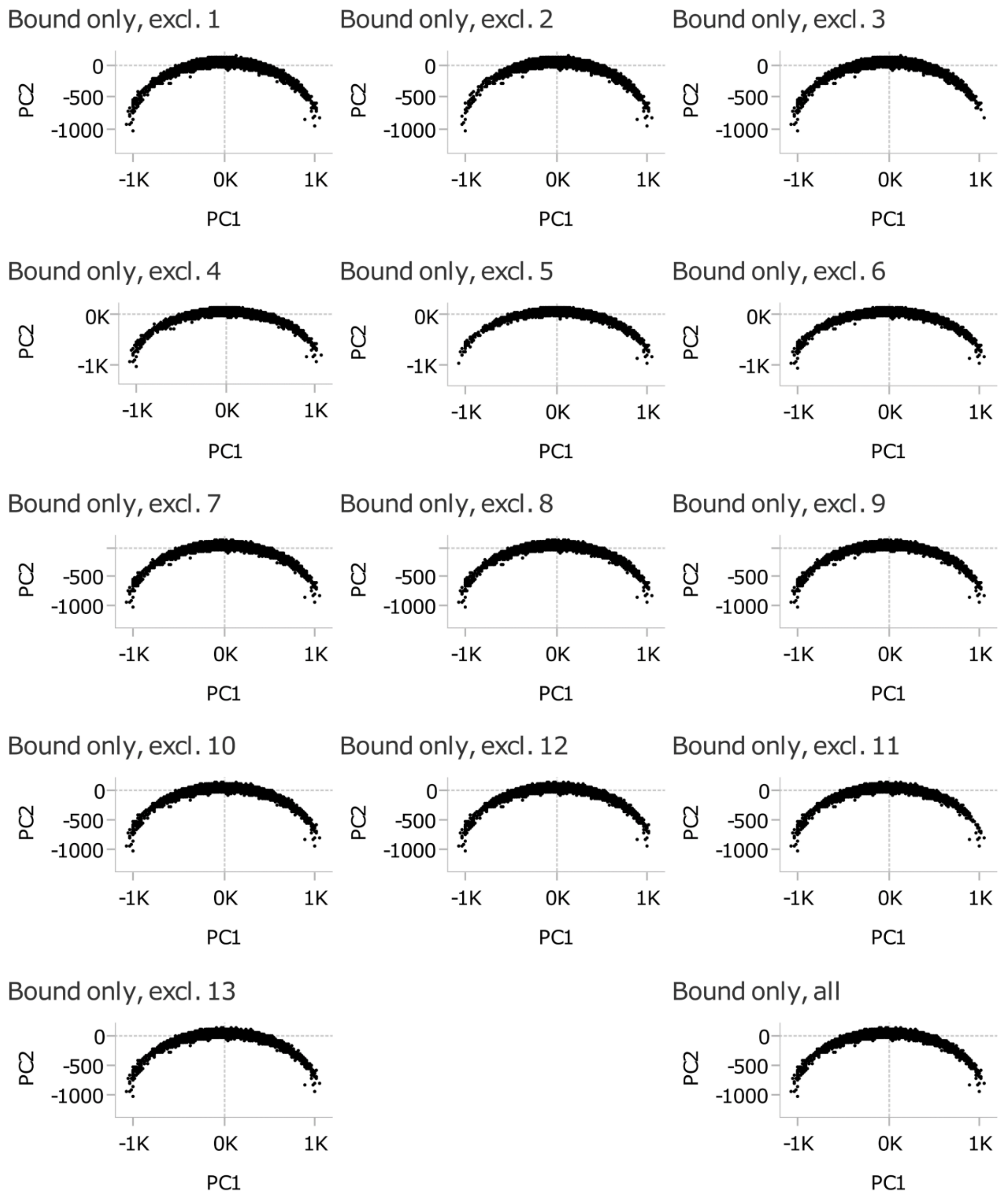

Fig. S2 PglB-NAT jack-knife (leave-one-out) analysis of first two principal components from Cartesian PCA excluding unstructured EL5 N-terminal portion (residues 282-306), alignment on stable core.

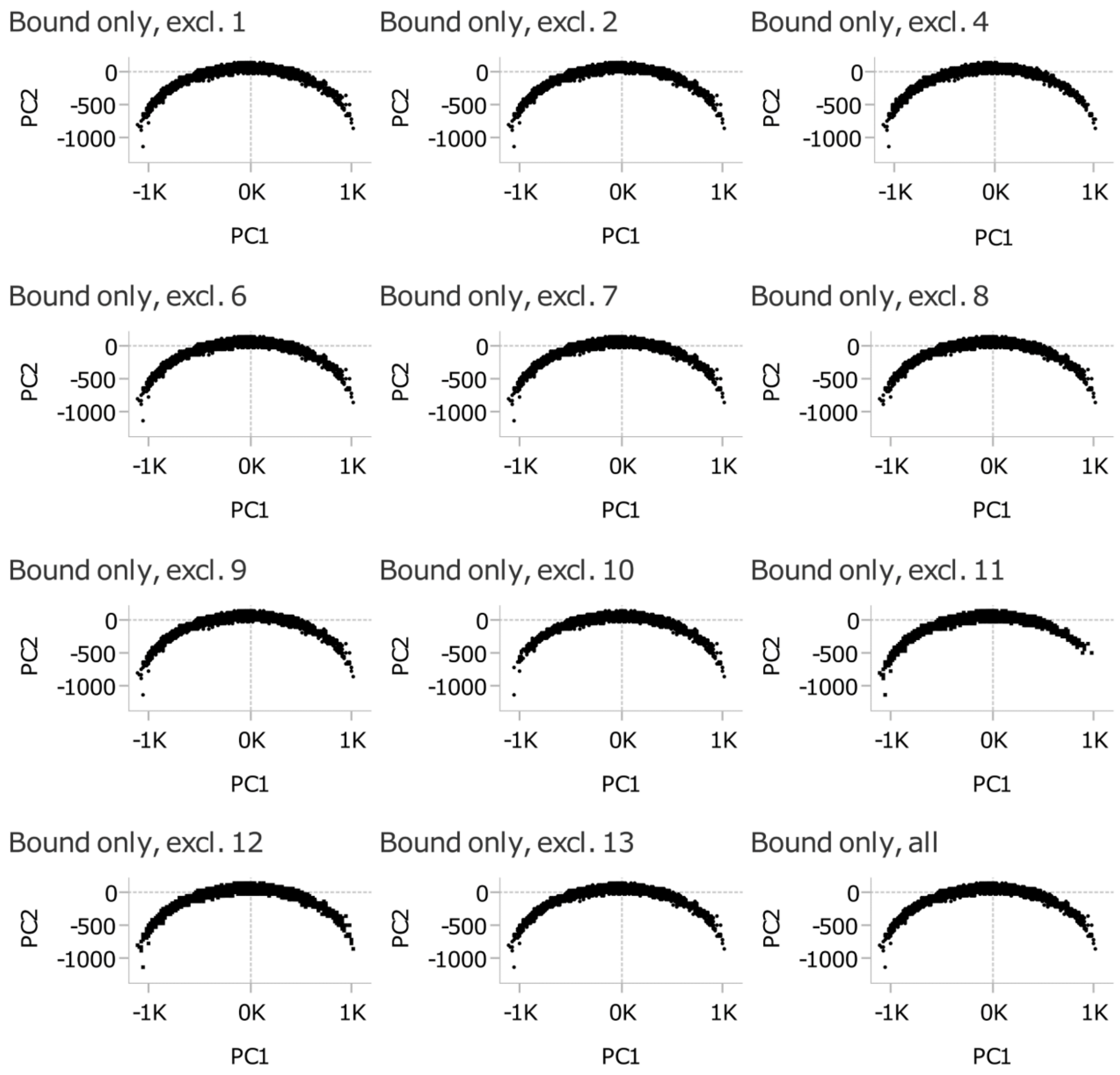

Fig. S3 PglB-NAS jack-knife (leave-one-out) analysis of first two principal components from Cartesian PCA excluding unstructured EL5 N-terminal portion (residues 282-306), alignment on stable core. Trajectories 3 and 5 are not included in the analysis because they initiate unbinding during the 50 ns structural equilibration period.

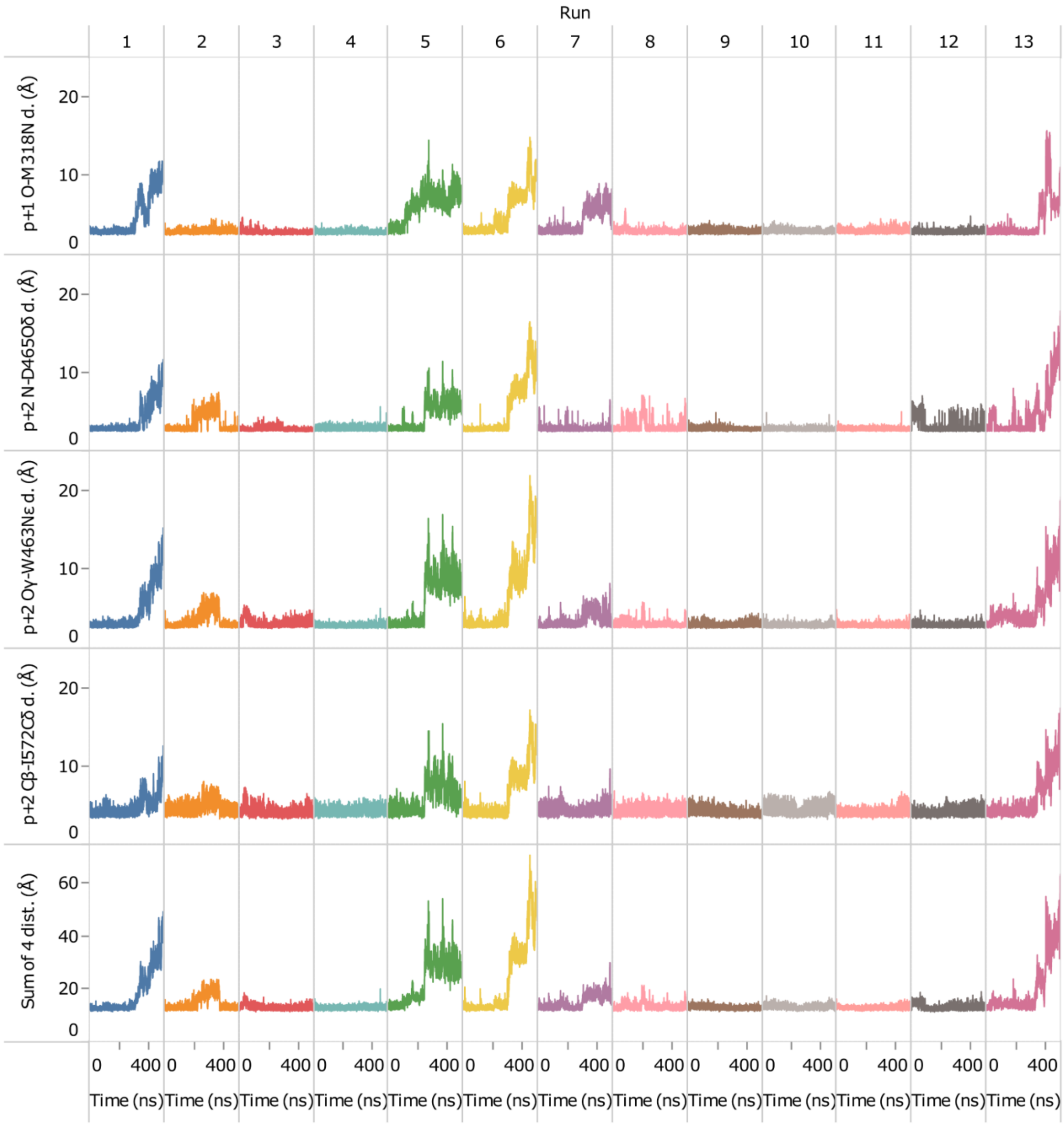

Fig. S4 PglB-NAT donor-acceptor distance of three non-catalytic H-bonds important for peptide binding (p+1 O-M318 N, p+2 N-D465 Oδ, and p+2 Oγ-W463 Nε), a proxy for the p+2-I572 van der Waals interaction (p+2 Cβ-I572 Cδ), and the sum of these four distances. Unbinding occurred at 334 ns (T1), 236 ns (T5), 299 ns (T6), and 344 ns (T13).

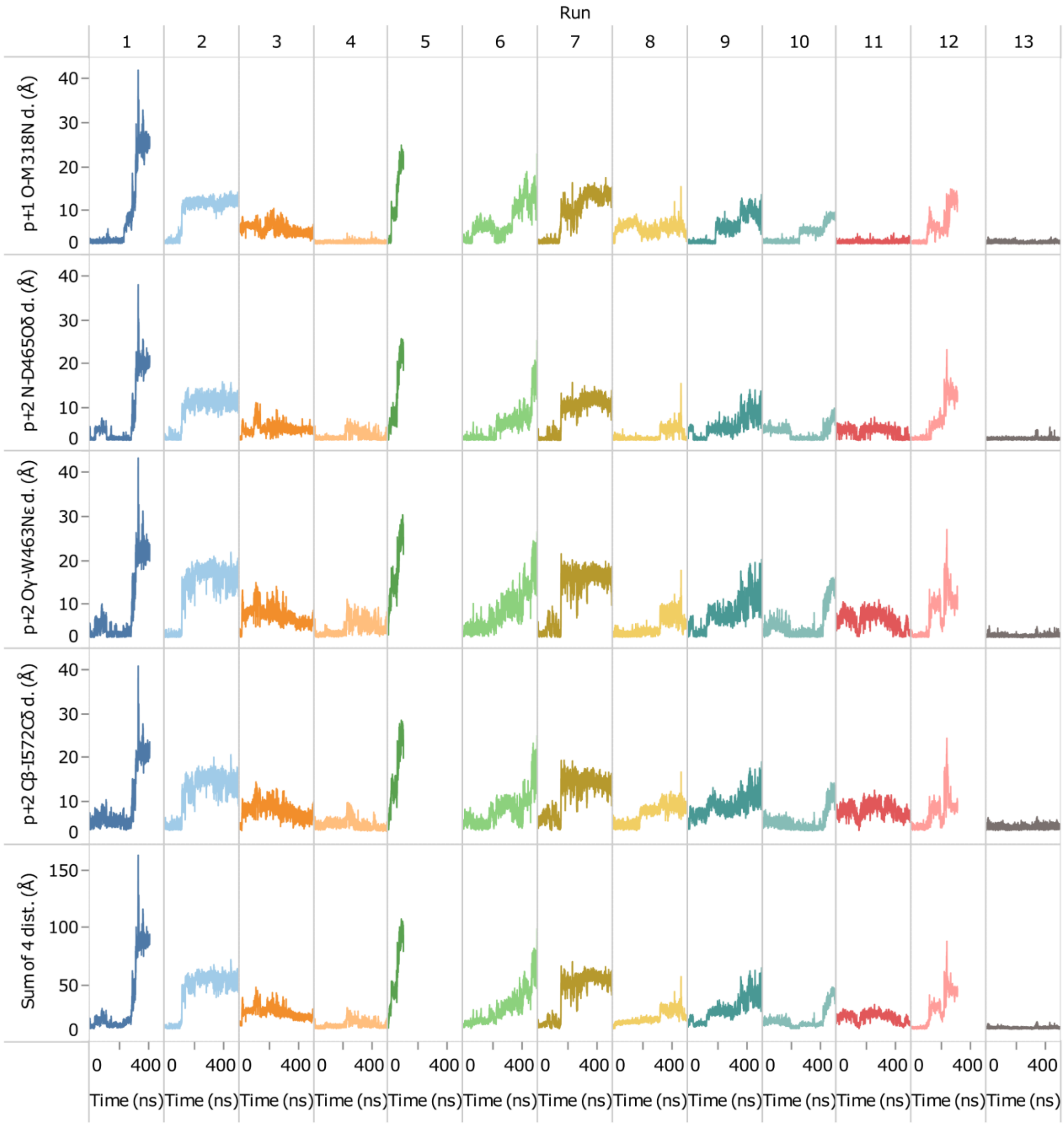

Fig. S5 PglB-NAS donor-acceptor distance of three non-catalytic H-bonds important for peptide binding (p+1 O-M318 N, p+2 N-D465 Oδ, and p+2 Oγ-W463 Nε), a proxy for the p+2-I572 van der Waals interaction (p+2 Cβ-I572 Cδ), and the sum of these four distances. Unbinding occurred at 276 ns (T1), 113 ns (T2), 14 ns (T3), 19 ns (T5), 252 ns (T6), 148 ns (T7), 307 ns (T8), 178 ns (T9), 401 ns (T10), and 155 ns (T12).

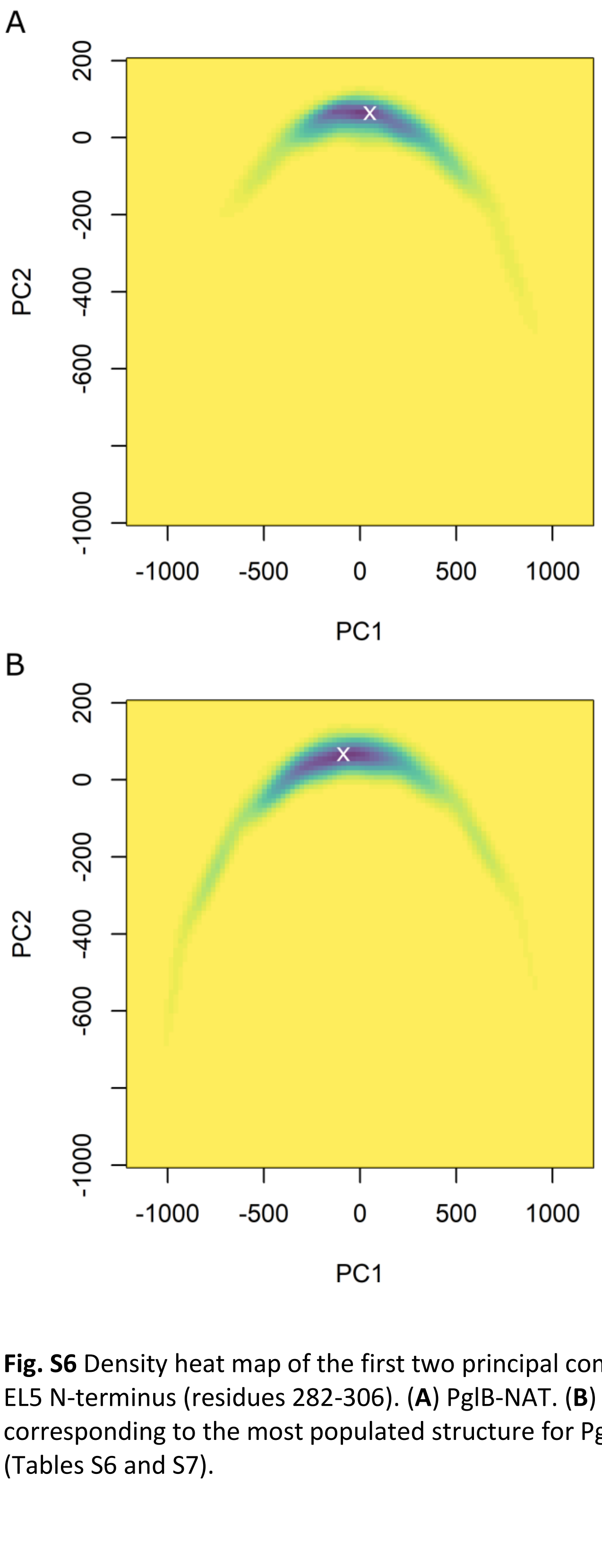

Fig. S6 Density heat map of the first two principal components (PC1 and PC2) excluding the unstructured EL5 N-terminus (residues 282-306). (**A**) PglB-NAT. (**B**) PglB-NAS. A white X marks the frame corresponding to the most populated structure for PglB-NAT and PglB-NAS used in the hinge analysis (Tables S6 and S7).

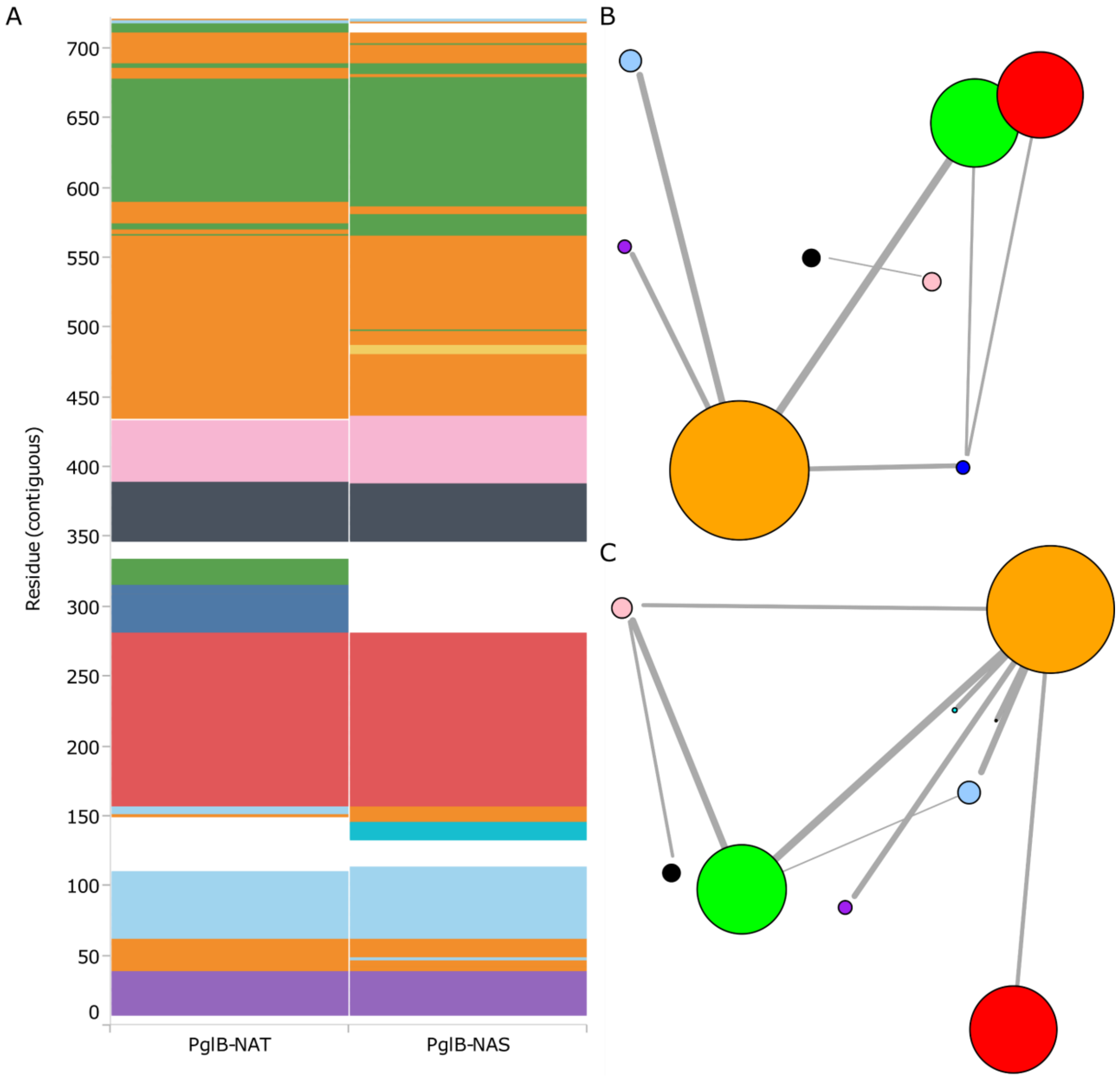

Fig. S7 Community partitioning by residue for PglB-NAT and PglB-NAS (**A**). Peptide residues are numbered 712-719, Mg^{2+} ions are numbered 720-721. Community colors correspond to coloring in the network graphs. Residues that are not connected to other communities are colored white. Simplified network graphs for PglB-NAT **(B)** and PglB-NAS **(C)** pruned of unconnected communities.

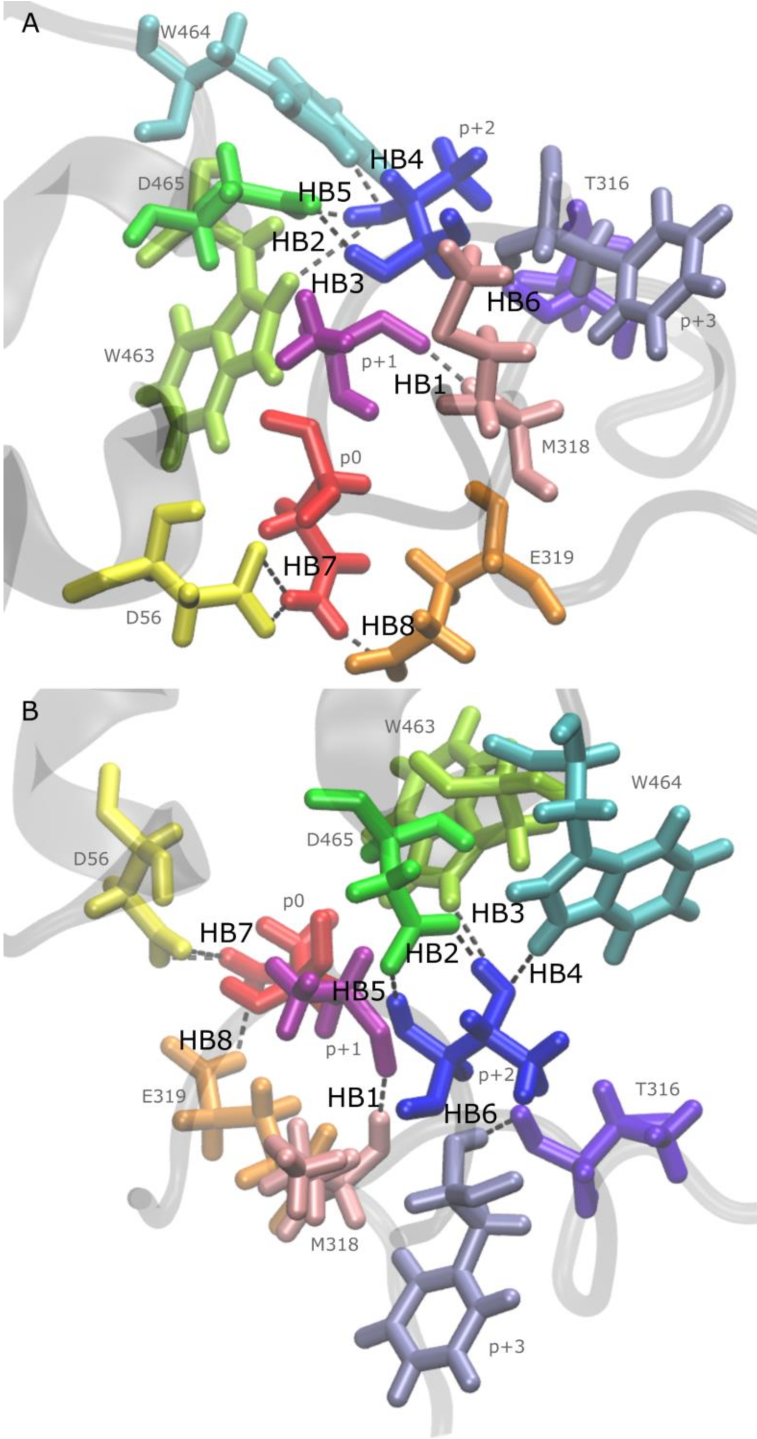

C

Label	H-bond
HB1	p+1 O-M318 N
HB2	p+2 N-D465 Oδ
HB3	p+2 Oγ-W463 Nε
HB4	p+2 Oγ-W464 Nε
HB5	p+2 Oγ-D465 Oδ
HB6	p+3 N-T316 O
HB7	p0 Nδ-D56 Oδ
HB8	p0 Nδ-E319 Oε

Fig. S8 Key hydrogen bonds. Hydrogen bond criteria is a donor-acceptor distance < 3.5 Å and a donor-hydrogen-acceptor angle of 130° < θ < 180°. (**A, B**) Front and side view of key hydrogen bonding pairs between peptide and PglB (NAT peptide shown, backbone of peptide and adjacent PglB residues in transparent gray): p0 (red), D56 (yellow), E319 (orange), T316 (violet), M318 (light pink), p+3 (lavender), p+1 (dark pink), p+2 (blue), W463 (lime), W464 (cyan), and D465 (green). (**C**) Table with H-bond label key.

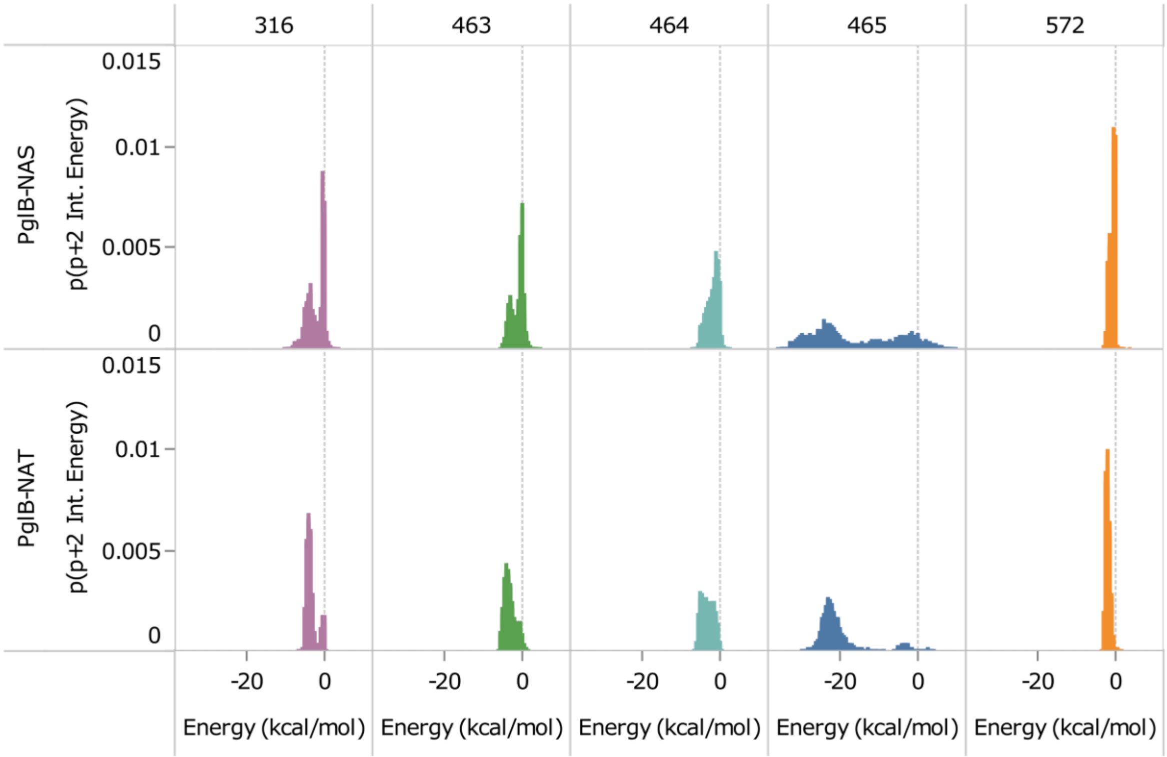

Fig. S9 p+2 interaction energy with PglB residues for the subset of residues where the absolute value of the difference in interaction energy is ≥ 1.0 kcal/mol for PglB-NAS and PglB-NAT.

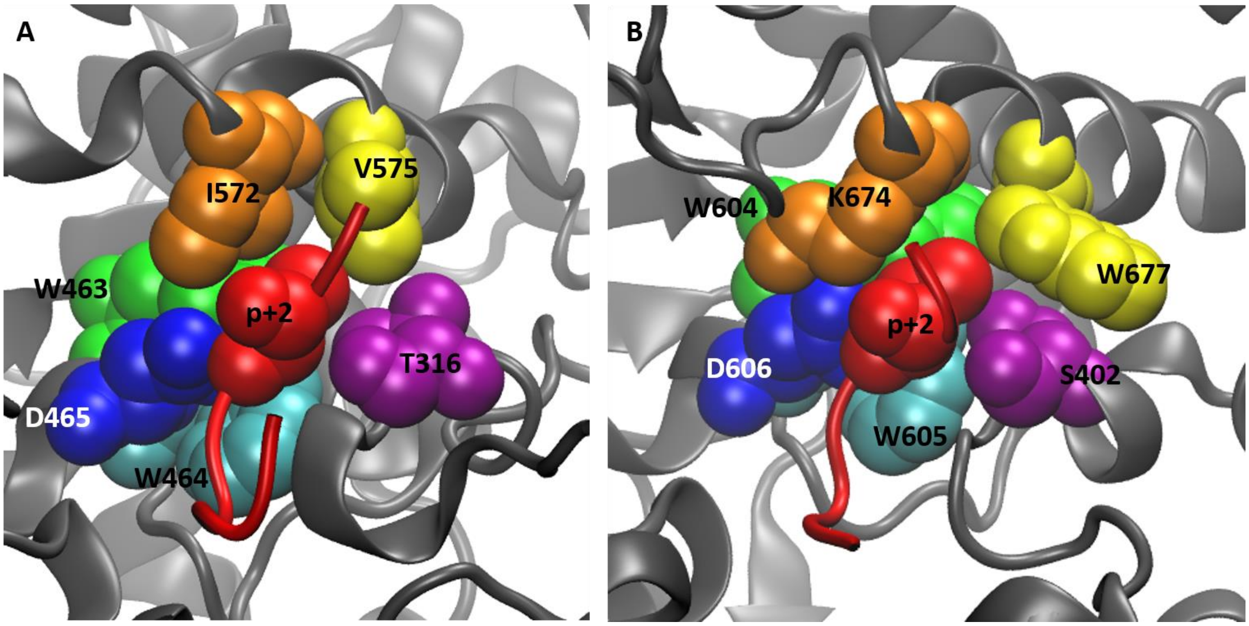

Fig. S10 Comparison of p+2 binding pocket in the crystal structure of *C. lari* PglB (**A**) and the cryo-EM structure of human OST-B STT3 subunit (**B**). Structurally analogous residues are colored the same in the two structures. Red: p+2, orange: I572/K674, yellow: V575/W677, green: W463/W604, cyan: W464/W605, blue: D465/D606, purple: T316/S402.
